# Advances in non-dopaminergic treatments for Parkinson's disease

**DOI:** 10.3389/fnins.2014.00113

**Published:** 2014-05-22

**Authors:** Sandy Stayte, Bryce Vissel

**Affiliations:** ^1^Neuroscience Department, Neurodegenerative Disorders Laboratory, Garvan Institute of Medical Research, SydneyNSW, Australia; ^2^Faculty of Medicine, University of New South Wales, SydneyNSW, Australia

**Keywords:** Parkinson's disease, animal models, therapeutics, neurodegeneration, L-Dopa, dyskinesias, dopamine, gene therapy

## Abstract

Since the 1960's treatments for Parkinson's disease (PD) have traditionally been directed to restore or replace dopamine, with L-Dopa being the gold standard. However, chronic L-Dopa use is associated with debilitating dyskinesias, limiting its effectiveness. This has resulted in extensive efforts to develop new therapies that work in ways other than restoring or replacing dopamine. Here we describe newly emerging non-dopaminergic therapeutic strategies for PD, including drugs targeting adenosine, glutamate, adrenergic, and serotonin receptors, as well as GLP-1 agonists, calcium channel blockers, iron chelators, anti-inflammatories, neurotrophic factors, and gene therapies. We provide a detailed account of their success in animal models and their translation to human clinical trials. We then consider how advances in understanding the mechanisms of PD, genetics, the possibility that PD may consist of multiple disease states, understanding of the etiology of PD in non-dopaminergic regions as well as advances in clinical trial design will be essential for ongoing advances. We conclude that despite the challenges ahead, patients have much cause for optimism that novel therapeutics that offer better disease management and/or which slow disease progression are inevitable.

## Introduction

As the life expectancy in industrialized countries increases, the burden of Parkinson's disease (PD) and the associated economic costs continues to rise, resulting in a dramatic need for effective treatments. Since the 1960's, treatments have been wholly symptomatic, involving a range of approaches to effectively restore, mimic, or replace dopamine (DA). While this treatment strategy, primarily through the use of levodopa (L-Dopa), still remains the most effective method of alleviating the symptoms of PD, its effectiveness is limited as long-term use is associated with the development of debilitating hyperkinetic movements including chorea, dystonia and athetosis, collectively known as L-Dopa-induced dyskinesias (LIDs). It is apparent therefore that the identification of alternative strategies is crucial.

**Figure 1:**
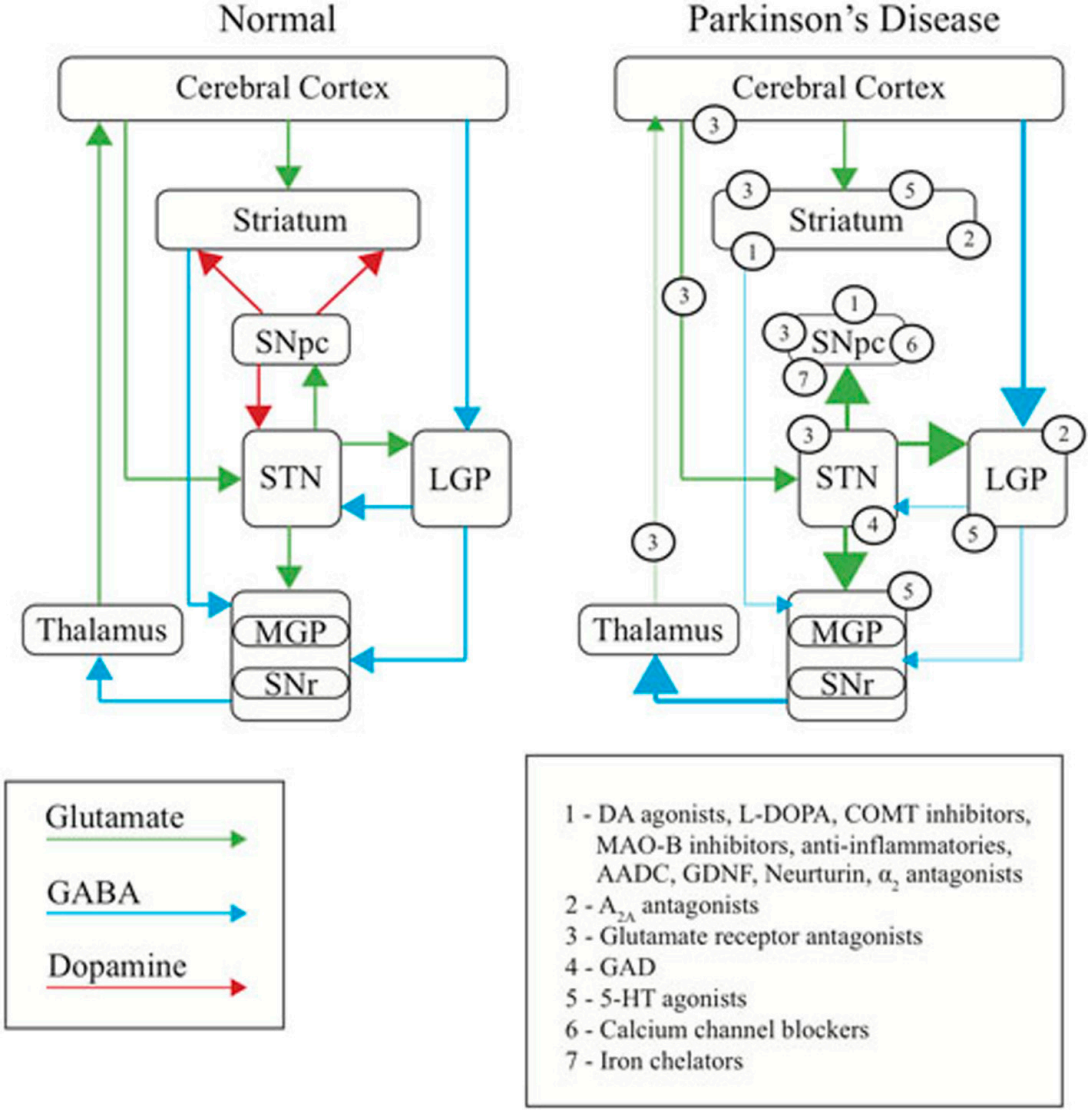
**Basal ganglia dysfunction in PD.** Diagram representing the normal function of the basal ganglia (**left**), the changes occurring in PD (**right**), and the site of primary action of therapeutic targets discussed in this review (numbered). Arrows represent the major neurotransmitters of glutamate (green), GABA (blue) and dopamine (red). Relative thickness of the arrows indicates level of activity of neurotransmitter. SNpc, substantia nigra pars compacta; SNr, substantia nigra reticulata; STN, subthalamic nucleus; MGP, medial globus pallidus; LGP, lateral globus pallidus.

The early success in developing treatment strategies relied on the early understanding that PD is a DA deficiency disorder. However, until recently this concept in many ways also constrained therapeutic development to a strategy of restoring or replacing DA signaling. Studies in rodents and in non-human primates have, however, more recently led to new insights into the mechanisms underlying PD. Such studies, together with some early studies of the effects of brain surgery in humans (Kumar et al., 1998; Burchiel et al., 1999), have been instrumental in subsequently redefining the motor symptoms of PD as the result of an imbalance of excitatory/inhibitory drive in the direct and indirect pathways of the basal ganglia (BG) (Albin et al., 1989, 1995; Graybiel, 1990; Gerfen, 1992; Porter et al., 1994; Wullner et al., 1994; Blandini et al., 2000; Wu et al., 2012) rather than simply resulting from a depletion of DA in the striatum (Dauer and Przedborski, 2003). This in turn has led to a shift in therapeutic development strategies away from DA and toward approaches that work in novel ways to restore the balance of BG signaling (Figure 1).

As this review will show, there has been progress. This has resulted in large part due to the ability of animal models to replicate changes in human BG circuits and in turn, provide valuable tools for testing therapies that work to restore the balance of excitatory/inhibitory drive. Animal models of PD have almost exclusively utilized various toxins such as MPTP, 6-OHDA, reserpine and the pesticide/herbicides paraquat and maneb, to reproduce the loss of DA neurons that, in turn, leads to altered signaling in the direct and indirect pathways of the BG. The reader is referred to a number of excellent reviews on rodent and non-human primate models of PD (Corasaniti et al., 1998; Przedborski and Vila, 2003; Przedborski et al., 2004; Smeyne and Jackson-Lewis, 2005; Simola et al., 2007; Thrash et al., 2007; Blandini et al., 2008; Duty and Jenner, 2011; Jackson-Lewis et al., 2012). Despite their well-known limitations (Beal, 2010; Potashkin et al., 2011), these animal models remain the standard for preclinical testing of novel therapeutics.

In turn, clinical trials have traditionally focused on testing novel treatment strategies arising from studies in animal models, addressing the fundamental movement disorders associated with PD. As we will show throughout this review, efficacy of these treatments is generally measured by the Unified Parkinson's Disease Rating Scale (UPDRS), Abnormal Involuntary Movements Scores (AIMs) as a measure of dyskinesia severity, and clinical and at home measurement of time spent moving freely versus period of time spent when medication is not working well and symptoms are not well controlled i.e., “on/off” time. To some extent these measures are limiting, as they are, at very least, subjective. More recently, there have been efforts to develop biomarkers of PD that may also act as markers of therapeutic benefit, however, these are in the earliest stages of development (Lewitt et al., 2013; Lin et al., 2013; Mollenhauer et al., 2013; Parnetti et al., 2013). While clinical trials continue to face significant challenges, perhaps in part resulting from the limitations of trial design (elaborated in section Clinical trials), newly discovered therapeutic approaches have in some cases resulted in encouraging outcomes.

In this review we aim to comprehensively assess the emerging non-dopaminergic pharmacological treatments of PD. We focus on the recent successes in translating outcomes of preclinical studies in animal models to clinical trials. The outcome is to show that there has been progress in identifying novel treatments to treat PD motor symptoms and LIDs. Meanwhile, as we will also discuss, efforts directed to understand the degenerative process and identify neuroprotective therapies in animal models is showing slower progress in translating preclinical results to positive outcomes in humans, potentially reflecting our poorer understanding of the mechanisms that underpin degeneration.

A summary of treatments that are directed to restore or replace dopamine such as L-Dopa, DA agonists, monoamine oxidase B inhibitors, continuous L-Dopa administration strategies, as well as the use of anticholinergics can be found in Table 1. However, while they continue to be of great significance as the mainstay of PD treatment, this review does not further discuss these therapies, as these have been extensively reviewed elsewhere (Hauser, 2009; Miyasaki, 2010; Perez-Lloret and Rascol, 2010; Schapira, 2011; Marsala et al., 2012; Sprenger and Poewe, 2013).

**Table 1:** **Current treatment strategies in clinical use**.

**Drug class**	**Drug name/route**	**Clinical use**	**Advantages**	**Disadvantages**
Levodopa (with Dopa Decarboxylase inhibitors)	Sinemet, Parcopa, Atamet	Monotherapy	Increase levels of endogenous DA	Motor fluctuations
				Dyskinesias
COMT Inhibitors	Entacapone, Tolcapone	Adjunct therapy	Decrease metabolism of L-Dopa	Dyskinesias
			Decrease in daily dose of L-Dopa required	Diarrhea
			Increase daily “on” time and UPDRS scores	Hepatic toxicity (tolcapone)
				Dizziness
				Insomnia
				Nausea
Dopamine agonists	Piribedil, Pramipexole, Pramipexole extended release, Ropinirole, Rotigotine, Cabergoline, Pergolide	Monotherapy (younger patients)	Increase levels of endogenous DA	Sedation
	Bromocriptine	Adjunct therapy	Decrease motor symptoms in early stages of disease	Impulse control disorder
				Somnolence
				Edema
Levodopa, Carbidopa, Entacapone combination	Stalevo	Monotherapy	Increase levels of endogenous DA	Dyskinesias may appear sooner
			Decrease metabolism of L-Dopa	Side effects are the same as for individual drug classes
MAO-B Inhibitors	Rasagiline, Selegiline, Safinamide	Initial monotherapy (mild PD patients)	Decrease catabolism of DA	Generally well tolerated
		Adjunct therapy	Decrease rate of progression on UPDRS	Mild nausea
				Constipation
				Confusion
Continuous L-Dopa	Intravenous bolus, Intravenous infusion, Intestinal carbidopa/L-Dopa gel	Monotherapy	Decrease pulsatile DA levels	Large volumes required (intravenous)
			Increase control of on/off periods	Requires surgery and prosthetic device
			Decrease dyskinesia severity and duration	Mechanical problems
			Decrease non motor symptoms (eg mood shifts, dribbling and urinary function changes)	Gastronomy complications
Anticholinergics	Trihexyphenidyl, Benztropine, Orphenadrine, Procyclidine, Biperiden	Monotherapy	Decrease acetylcholine levels	Dry mouth
		Adjunct Therapy	Decrease tremor	Blurred vision
				Constipation
				Nausea
				Impaired sweating

Abbreviations: COMT, catechol-O-methyl transferase; DA, dopamine; L-Dopa, levodopa; MAO-B, monoamine oxidase B; UPDRS, Unified Parkinson's Disease Rating Scale.

**Table 2:** **Effects of non-dopaminergic therapies in animal models**.

**Drug class**	**Subclass**	**Effect in animal models**	**References**
Adenosine receptor antagonists	A_2A_ antagonists Preladenant, Istradefylline, SCH58261, SCH412348, MSX-3	↓ catalepsy in reserpine and haloperidol models	Mandhane et al., 1997; Kanda et al., 1998; Shiozaki et al., 1999; Ikeda et al., 2002; Salamone et al., 2008; Hodgson et al., 2009; Trevitt et al., 2009
		↑ L-Dopa-induced contralateral turning behavior in 6-OHDA model	
		↓ behavioral sensitization induced by L-Dopa	
		↑ locomotion in MPTP and reserpine models	
		↑ survival of DA neurons in 6-OHDA model	
		↓ striatal DA nerve terminal loss and gliosis in MPTP model	
	Non-specific Caffeine, Theophylline, DMPX	↓ catalepsy in reserpine and haloperidol models	Mandhane et al., 1997; Chen et al., 2001; Xu et al., 2002; Bishnoi et al., 2006; Kalda et al., 2006; Singh et al., 2009; Trevitt et al., 2009; Kachroo et al., 2010
		↑ survival of DA neurons in MPTP, paraquat and maneb models	
NMDA Receptor Antagonists	NMDA Antagonists MK-801, Amantadine, Memantine, L-701,324, SDZ 220-58, Dextromethorphan, CPP	↓ haloperidol-induced catalepsy	Carlsson and Carlsson, 1989; Graham et al., 1990; Mehta and Ticku, 1990; Loschmann et al., 1991; Turski et al., 1991; Zuddas et al., 1992a,b; Lange et al., 1993; Srivastava et al., 1993; Kaur and Starr, 1995; St-Pierre and Bedard, 1995; Marin et al., 1996; McAllister, 1996; Kaur et al., 1997; Konieczny et al., 1999; Dutra et al., 2002; Kelsey et al., 2004; Armentero et al., 2006
		↓ akinesia in reserpine model	
		↓ parkinsonian symptoms in MPTP model	
		↑ stepping with contralateral paw in 6-OHDA model	
		↑ contralateral circling in 6-OHDA model	
		↓ dyskinesias in MPTP and 6-OHDA models	
		↑ DA neuron survival, DA levels in MPTP and 6-OHDA models	
		↑ efficacy of L-Dopa	
	NMDA-NR2B antagonists Ifenprodil, Traxoprodil, Co101244	↓ parkinsonian symptoms in MPTP model	Blanchet et al., 1999; Nash et al., 2000; Steece-Collier et al., 2000
		↑ efficacy of L-Dopa	
		↓ haloperidol-induced catalepsy	
		↓ appearance of LIDs	
AMPA Receptor Antagonists	NBQX, Perampanel, Talampanel	↑ contralateral rotations in 6-OHDA model	Klockgether et al., 1991; Loschmann et al., 1991, 1992; Wachtel et al., 1992; Konitsiotis et al., 2000
		↓ reserpine-induced muscle rigidity	
		↑ L-Dopa-induced reversal of motor deficits in MPTP and 6-OHDA models	
		↓ LIDs in MPTP model	
		↓ L-Dopa-induced AIMs	
	Topiramate	↓ LIDs in MPTP model	Silverdale et al., 2005; Kobylecki et al., 2011
		↓ L-Dopa-induced AIMs in 6-OHDA model	
		↓ rotarod performance in 6-OHDA model	
Metabotropic Glutamate Receptor Antagonists	Group I Metabotropic	↓ akinesia in 6-OHDA model	Kearney et al., 1997, 1998; Breysse et al., 2002, 2003; Coccurello et al., 2004; Kachroo et al., 2005; Vernon et al., 2005, 2007; Dekundy et al., 2006; Rylander et al., 2009; Price et al., 2010; Morin et al., 2013a,b
	Glutamate Receptor Antagonists MPEP, MTEP, LY367386, Dipraglurant, AFQ056	↓ LIDs in 6-OHDA model	
		↑ survival of DA neurons in 6-OHDA model	
		↑ striatal DA in 6-OHDA model	
Serotonin Receptor Agonists	5-HT_1A_ Agonists	↓ LIDs in 6-OHDA and MPTP models	Carta et al., 2007; Munoz et al., 2008; Lindgren et al., 2010
	8-OH-DPAT	↓ activity of raphe-striatal neurons and extracellular DA release in 6-OHDA model	
	5-HT_1B_ Agonists	↓ LIDs in 6-OHDA model	Carta et al., 2007; Lindgren et al., 2010
	CP-94253	↓ activity of raphe-striatal neurons and extracellular DA release in 6-OHDA model	
	5-HT_1A/1B_ Agonists	↓ LIDs in 6-OHDA model	Bezard et al., 2013
	Eltoprazine	↓ LIDs in MPTP model	
		↓ LIDs in combination with amantadine	
Adrenergic Receptor Antagonists	α2 Receptor Antagonists Yohimbine, Idazoxan, Fipamezole	↓ L-Dopa-induced hyperkinesia in 6-OHDA model	Gomez-Mancilla and Bedard, 1993; Henry et al., 1998, 1999; Fox et al., 2001; Invernizzi et al., 2003; Savola et al., 2003; Johnston et al., 2010; Barnum et al., 2012
		↓ expression of AIMs in 6-OHDA model	
		↓ haloperidol-induced catalepsy	
		↓ LIDs in MPTP model	
		↑ “on time” and “on time without disabling dyskinesia” in MPTP model	
Calcium Channel Blockers	Cav1.3 Channel Blockers Isradipine	↑ survival of DA nigral neurons in MPTP and 6-OHDA models	Chan et al., 2007, 2010; Schuster et al., 2009; Ilijic et al., 2011; Kang et al., 2012
		↓ degeneration of striatal DA fibers in 6-OHDA model	
		↓ development of motor deficits in MPTP model	
		↓ LIDs and AIMS in 6-OHDA model	
Glucagon-like Peptide 1 Agonists	GLP-1 Analogs Exendin-4	↑ survival of DA neurons, DA levels in MPTP model	Harkavyi et al., 2008; Li et al., 2009
		↑ motor function in MPTP model	
		↑ rescue of DA neurons, DA levels and apomorphine-induced circling in 6-OHDA and LPS models	
Iron Chelators	Chelators Desferrioxamine, VK-28, Clioquinol, M30, Deferiprone	↑ striatal DA and normal motor behavior in 6-OHDA model	Ben-Shachar et al., 1991, 1992; Lan and Jiang, 1997; Kaur et al., 2003; Shachar et al., 2004; Youdim et al., 2004; Youdim, 2012; Gal et al., 2005
		↑ survival of DA neurons and striatal DA content in MPTP model	
Anti-inflammatories	Non-selective NSAIDs Aspirin, Salicylic acid, Indomethacin	↑ survival of DA neurons in MPTP model	Aubin et al., 1998; Ferger et al., 1999; Kurkowska-Jastrzebska et al., 2002
		↑ striatal DA in MPTP model	
		↓ microglial activation and lymphocyte infiltration in MPTP model	
	Selective COX-2 inhibitors Meloxicam, Celocoxib	↑ survival of DA neurons in 6-OHDA and MPTP models	Teismann and Ferger, 2001; Sanchez-Pernaute et al., 2004
		↑ striatal DA in MPTP model	
		↑ locomotor activity in MPTP model	
		↓ microglial activation in 6-OHDA model	
Gene Therapy	Non-Growth Factors AADC, GAD	↑ DA levels	Fan et al., 1998; Leff et al., 1999; Sanchez-Pernaute et al., 2001; Luo et al., 2002; Lee et al., 2005; Emborg et al., 2007
		↑ behavioral recovery in 6-OHDA model	
		↑ survival of DA neurons and DA levels in 6-OHDA model	
		↓ rotation rates in 6-OHDA model	
		↑ improvement in bradykinesia, gross motor skills, and tremor in MPTP model	
Neurotrophic Factors	GDNF	↑ density of TH positive fibers and DA levels in 6-OHDA and MPTP models	Sauer et al., 1995; Tomac et al., 1995; Miyoshi et al., 1997; Zhang et al., 1997; Gerhardt et al., 1999; Kirik et al., 2000a,b; Palfi et al., 2002; Wang et al., 2002; Eslamboli et al., 2003; Eberling et al., 2009; Johnston et al., 2009
		↑ survival of DA neurons and DA levels in 6-OHDA model	
		↓ parkinsonism and LIDs in MPTP model	
		↑ behavioral recovery in 6-OHDA and MPTP models	
	Neurturin	↑ survival of DA neurons and DA levels in 6-OHDA and MPTP models	Rosenblad et al., 1999; Oiwa et al., 2002; Kordower et al., 2006; Gasmi et al., 2007a,b; Herzog et al., 2007
		↑ density of TH positive fibers	
		↑ motor function in MPTP model	

Abbreviations: AMPA, α-amino-3-hydroxy-5-methyl-4-isoxazolepropionic acid; 6-OHDA, 6-hydroxydopamine; MPTP, 1-methyl-4-phenyl-1,2,3,6-tetrahydropyridine; AIMs, abnormal involuntary movements; AADC, aromatic l-amino acid decarboxylase; DA, dopamine; GDNF, glial derived neurotrophic factor; GAD, glutamic acid-decarboxylase; LIDs, L-Dopa-induced dyskinesias; L-Dopa, levodopa; LPS, lipopolysaccharide; NMDA, N-methyl-D-aspartate; NSAIDs, non-steroidal anti-inflammatory drugs; TH, tyrosine hydroxylase.

**Table 3:** **Effects of non-dopaminergic therapies in human clinical trials**.

**Drug class**	**Drug name**	**Clinical trial results**	**Status**	**References**
Adenosine A_2A_ receptor antagonists	Istradefylline (KW-6002)	Effects	Phase III in progress	Bara-Jimenez et al., 2003; Hauser et al., 2008; Lewitt et al., 2008; Stacy et al., 2008
		↑ time spent “on”		
		↓ time spent “off”		
		Adverse events		
		↑ time spent “on” with dyskinesias		
	Preladenant	Effects	Phase III recruiting	Hauser et al., 2011
		↓ time spent “off”		
		↑ time spent “on” with dyskinesias		
		↑ time spent “on” with non-troublesome dyskinesias		
		Adverse events		
		↑ time spent “on” with troublesome dyskinesias		
NMDA antagonists	Amantadine	Effects	Used in the clinic as anti-dyskinetic treatment	Verhagen Metman et al., 1998
		↓ dyskinesia severity		
		↓ motor fluctuations		
		Benefits may decrease over time		
	Memantine	Effects	Phase IV in progress	Emre et al., 2010; Varanese et al., 2010; Moreau et al., 2013
		↑ cognitive scores in dementia with Lewy bodies	Approved for other conditions (Alzheimer's disease)	
		↑ improvement of LIDs		
		↑ improvement in on/off timing		
		↓ axial motor symptom scores		
AMPA receptor antagonists	Perampanel	Effects	Phase II complete	Eggert et al., 2010; Lees et al., 2012
		Well tolerated and safe	Discontinued/withdrawn	
		Failure to improve “wearing off” times		
	Talampanel	More results pending completion of study and publication	Phase II complete	Clinicaltrials.gov reference numbers
				NCT00036296 NCT00108667 NCT00004576
	Topiramate	Two Phase II trials terminated due to poor recruitment or cessation of funding	Phase II recruiting	Clinicaltrials.gov reference numbers
		Known Adverse Events		NCT00794313
		Depression, aggressive behavior, irritability, psychosis		NCT00296959
				NCT01789047
Metabotropic glutamate receptor antagonists (Group 1)	Dipraglurant	Effects	Phase II in progress	Clinicaltrial.gov reference number
		↓ dyskinesia severity		NCT01336088
		↑ time “on” without dyskinesia		
		↓ time “off”		
		Adverse events		
		Vertigo, visual disturbances, “feeling drunk”		
	AFQ056	Effects	Phase II recruiting	Berg et al., 2011
		↓ LFADLDS scores		Clinicaltrial.gov reference numbers
		↓ AIMs		
		Adverse events		NCT01173731
		Worsening of dyskinesias, dizziness, fatigue		NCT01491529
Serotonin receptor agonists (5-HT1A)	Sarizotan	Effects	Phase II completed	Olanow et al., 2004; Bara-Jimenez et al., 2005; Goetz et al., 2007
		↓ dyskinesias as measured by AIMS and UPDRS scores	Discontinued/withdrawn	
		↑ improvement in “off” time		
		Adverse events		
		↑ parkinsonism		
	Buspirone	Effects	Phase II completed	Bonifati et al., 1994
		↓ severity of dyskinesias		
	Mirtazapine	Effects	Phase II completed	Meco et al., 2003
		↓ LIDs		
	Clozapine	Effects	Approved for other conditions	Durif et al., 1997; Kannari et al., 2002; Durif et al., 2004; Meco et al., 2009
	Tandosprione Aripiprazole	↓ dyskinesias		
Adrenergic receptor antagonists	Idazoxan	Effects	Phase II completed	Rascol et al., 2001
		↓ severity of LIDs		
	Fipamezole	Effects	Phase II completed	Lewitt et al., 2012
		↓ LIDs		Clinicaltrials.gov reference numbers
		Adverse events		
		↑ blood pressure		NCT01149811
				NCT00040209
				NCT01140841
Calcium channel blockers	Isradipine	Effects	Phase II recruiting	Rodnitzky, 1999; Simuni et al., 2010; Rees et al., 2011
		↓ expected incidence of PD	Approved for other conditions	
		Adverse events		
		↑ incidence of leg oedema		
		↓ blood pressure		
		Dizziness		
Glucagon-like peptide 1 agonists	Exendin-4	Effects	Phase II completed	Aviles-Olmos et al., 2013
		↑ motor function		
		↑ cognitive function		
		Adverse Events		
		Weight loss, distortion of taste, L-Dopa dose failure, increased parkinsonism		
Iron chelators	Deferiprone	Effects	Phase II in progress	Devos et al., 2014
		↓ iron accumulation in SN		Clinicaltrials.gov reference numbers
		↑ motor function		
		Adverse events		NCT01539837
		Transient diarrhea, gastritis, transient headache, slight proctorrhagia		NCT00907283
Anti-inflammatories	NSAIDS (non-aspirin)	Effects	Meta analysis. No formal clinical trial	Gagne and Power, 2010
		No formal clinical studies		
		↓ risk of developing PD based on epidemiological studies		
		Adverse events		
		↑ risk of gastrointestinal side effects		
	Minocycline	Effects	Phase II completed	NINDS NET-PD Investigators, 2006; NINDS NET-PD Investigators, 2008
		Passed futility threshold of 30% reduction in UPDRS	Approved for other conditions	
		No adverse effect on response to symptomatic therapy		
		Adverse events		
		Nausea, upper respiratory symptoms, joint pain, arthritis		
Gene therapy	AADC	Effects	Phase I complete	Christine et al., 2009; Muramatsu et al., 2010
		↑ off medication motor function		
		↑ AADC activity in striatum		
		Adverse events		
		Brain hemorrhage along catheter trajectory		
	GAD	Effects	Phase II complete	Kaplitt et al., 2007; Lewitt et al., 2011
		↑ improvement in UPDRS scores		
		↓ in UPDRS “off” scores		
	GDNF	Effects	Pump infusion− discontinued/withdrawn	Kordower et al., 1999; Nutt et al., 2003; Slevin et al., 2007
		No effect on primary outcomes when administered continuously	Gene therapy−Phase 1 recruiting	Clinicaltrial.gov reference number
		Many trials using AAV-GDNF still ongoing		NCT01621581
		Adverse events		
		Paresthesias, asymptomatic hyponatremia		
	Neurturin (CERE-120)	Effects	Phase II complete	Marks et al., 2008, 2010; Bartus et al., 2011, 2013
		↓ in UPDRS “off” scores		
		↑ “on” time without dyskinesias		
		↓ total “off” time		
		Deficits in retrograde transport of CERE-120 when administered to putamen No effect on primary outcomes when administered in dual locations		

Abbreviations: AMPA, α-amino-3-hydroxy-5-methyl-4-isoxazolepropionic acid; AIMs, abnormal involuntary movements; AAV, adeno-associated virus; AADC, aromatic l-amino acid decarboxylase; GDNF, glial derived neurotrophic factor; GAD, glutamic acid-decarboxylase; LFADLDS, Lang-Fahn Activities of Daily Living Dyskinesia Scale; LIDs, L-Dopa-induced dyskinesias; L-Dopa, levodopa; NMDA, N-methyl-D-aspartate; NSAIDs, non-steroidal anti-inflammatory drugs; PD, Parkinson’s disease; UPDRS, Unified Parkinson’s Disease Rating Scale.

### Adenosine receptor antagonists

Adenosine is a neuromodulator that regulates responses to DA and other neurotransmitters in areas of the brain that are responsible for motor function and learning and memory (Latini and Pedata, 2001). Of the four subtypes of adenosine receptors, the A_2A_ subtype is densely localized in the BG, with the greatest density found in the striatum. These receptors have been shown to be co-localized with D2 receptors on enkephalin-expressing output neurons of the indirect pathway or found as A_2A_-D2 heteromers (Hettinger et al., 2001; Ishiwata et al., 2005; Jenner et al., 2009; Soriano et al., 2009) and are therefore thought to play an important role in the regulation of dopaminergic transmission in the BG. Postmortem studies of PD patients have demonstrated a 2.95-fold increase in A_2A_-receptor expression in the putamen compared to healthy subjects and increased levels in dyskinetic patients treated with L-Dopa compared to L-Dopa-treated patients that displayed no dyskinesias (Calon et al., 2004; Varani et al., 2010).

There have been numerous studies utilizing animal models of PD to investigate A_2A_-receptor antagonists as viable therapeutics. The A_2A_ antagonists caffeine, theophylline, SCH58261, DMPX and KF17837 were all shown to inhibit motor deficits such as catalepsy and decreased locomotion induced by haloperidol and in models of tardive dyskinesia in rodents (Mandhane et al., 1997; Bishnoi et al., 2006, 2007; Salamone et al., 2008; Trevitt et al., 2009). It has also been shown that oral administration of preladenant and SCH412348 potentiated L-Dopa-induced contralateral rotation behavior in animals lesioned with 6-OHDA.

Further, daily administration of the A_2A_-receptor antagonist preladenant inhibited behavioral sensitization induced by L-Dopa, suggesting that preladenant may reduce the risk of the development of dyskinesias (Hodgson et al., 2009). The use of istradefylline (KW-6002) has also been demonstrated to ameliorate the hypolocomotion induced by MPTP and reserpine and to also exert significant anti-cataleptic benefits in the haloperidol and reserpine models of PD when combined with administration of L-Dopa (Shiozaki et al., 1999). Furthermore, KW-6002 has demonstrated little or no induction of dyskinesias in L-Dopa-primed MPTP-treated marmosets (Kanda et al., 1998).

A_2A_ antagonists have also demonstrated a potential neuroprotective role in animal models. The use of caffeine *in vivo* has been shown to protect dopaminergic neurons in mice treated with the PD toxins MPTP, paraquat or maneb (Chen et al., 2001; Xu et al., 2002; Kalda et al., 2006; Singh et al., 2009; Kachroo et al., 2010). Although caffeine has been shown to act on both A_1_ and A_2A_ receptors, it has been suggested that its neuroprotective properties result primarily through its interaction with A_2A_, with the effects of caffeine largely abolished in A_2A_ receptor knockout mice (El Yacoubi et al., 2000; Huang et al., 2005). A_2A_ antagonists have also been demonstrated to protect against dopaminergic neuron loss in the substantia nigra (SN) induced by 6-OHDA in rats in addition to preventing the functional loss of striatal dopaminergic nerve terminals and gliosis as a result of MPTP treatment in mice (Ikeda et al., 2002).

Due to the promising results in animal models of PD, the adenosine A_2A_ receptor antagonist KW-6002 has been investigated in a number of human clinical trials. KW-6002 potentiated the effects of concomitant low dose L-Dopa treatment with an improvement in the amount of time spent “on” and no exacerbation of dyskinesias in a small Phase I study (Bara-Jimenez et al., 2003). Subsequently, two large, randomized, double-blind, placebo-controlled Phase II studies in advanced PD patients demonstrated significant reductions in the amount of time spent “off” over a 12 week period (Lewitt et al., 2008; Stacy et al., 2008; Mizuno and Kondo, 2013). These findings were replicated by a large Phase III trial in advanced PD patients where KW-6002 treatment resulted in an 0.7 h reduction in daily “off” times, sustained over 12 weeks, and also resulting in increased functional “on” time (Hauser et al., 2008). Furthermore, this reduction in “off” time was sustained over long time periods, with patients displaying improvements from baseline scores up to 1 year later (Factor et al., 2010). In most of the clinical trials KW-6002 treatment was associated with some increase in “on time with dyskinesias” and the presence of dyskinesias was reported as an adverse event more often in the KW-6002 groups. While an application for KW-6002 as a new PD drug was declined in the United States by the FDA in 2008 (Kyowa Hakko Kirin Co Ltd, 2008), KW-6002 was approved in March 2013 in Japan as an adjunct treatment to L-Dopa for PD (Kyowa Hakko Kirin Co Ltd, 2013).

The effect of the A_2A_ antagonist preladenant was also investigated in a 12 week Phase II clinical trial in PD patients experiencing motor fluctuations (Hauser et al., 2011). There was a significant reduction in “off” time, however, preladenant treatment also increased total “on” time with dyskinesias and “on” time with non-troublesome dyskinesias. Those receiving the highest dose of preladenant also reported an increase in the amount of time spent “on” with troublesome dyskinesia. A Phase II open-label follow up trial was then conducted in which patients received preladenant twice daily for 36 weeks to assess long-term safety and efficacy. The primary endpoint of adverse events was reported in 88% of patients, with dyskinesias and constipation the most common (Factor et al., 2013). Much like KW-6002, preladenant treatment does not appear to reduce dyskinesias, however, it remains to be determined if preladenant causes less dyskinesia than KW-6002.

### Glutamate receptor antagonists

There are two main classes of glutamate receptors, ionotropic and metabotropic. Ionotropic glutamate receptors, including the N-methyl-D-aspartate (NMDA), α-amino-3-hydroxy-5-methyl-4-isoxazolepropionic acid (AMPA) and Kainate subtypes, mediate the majority of fast excitatory transmission throughout the central nervous system (CNS) and are important for numerous brain functions (Hollmann and Heinemann, 1994; Dingledine et al., 1999; Niswender and Conn, 2010; Traynelis et al., 2010; Wiltgen et al., 2010; Wright and Vissel, 2012). The metabotropic glutamate receptors (mGluRs) are G protein-coupled receptors that are differentially expressed throughout the BG and consist of eight subtypes (Conn et al., 2005; Kuwajima et al., 2007). Unlike ionotropic glutamate receptors, mGluRs modify neuron membrane potential through modulating excitatory and inhibitory synaptic transmission by both pre- and postsynaptic mechanisms (Conn et al., 2005).

Whilst PD has long been known as a condition arising from a lack of DA-producing neurons, there is a concurrent abnormal release of glutamate in BG circuits, generally considered a secondary consequence of decreased DA levels. It is thought that neuronal loss in the SN and consequent striatal DA depletion leads to excessive inhibitory output from the globus pallidus internus (GPi) and substantia nigra pars reticulata (SNr). This is due to disinhibition of the subthalamic nucleus (STN), which drives the GPi and SNr via the release of glutamate (Blandini et al., 2000). Furthermore, evidence suggests that glutamate-mediated excitotoxicity may be a primary cause of dopaminergic neuron loss and therefore aberrant glutamate regulation may also contribute to neurodegeneration in PD (Choi, 1988; Koutsilieri and Riederer, 2007). Given these observations, attenuating excitatory drive through antagonizing glutamate receptors may provide a therapeutic strategy for PD.

#### NMDA receptors

NMDA receptors are a major subtype of ionotropic glutamate receptors that are implicated in synaptic plasticity and excitotoxicity (Vissel et al., 2001; Carroll and Zukin, 2002; Perez-Otano and Ehlers, 2005; Lau and Zukin, 2007; Hardingham and Bading, 2010; Paoletti et al., 2013). As NMDA receptors are expressed in neurons of the striatum and STN, excessive activity of the indirect pathway would lead to hyperactivity of these receptors. By inference, NMDA antagonists would be expected to reduce excessive activation of these receptors in the indirect pathway with potential therapeutic effects on PD symptoms including reducing the development and severity of LIDs. In addition, blocking these receptors may also reduce excitotoxic cell loss and thereby have disease-modifying actions.

Several NMDA antagonists have been shown to reverse haloperidol-induced catalepsy and muscle rigidity (Mehta and Ticku, 1990; McAllister, 1996; Kaur et al., 1997; Konieczny et al., 1999) and also to reverse akinesia and other motor disturbances in reserpine-treated rodents (Carlsson and Carlsson, 1989; Kaur and Starr, 1995; Dutra et al., 2002). These effects have also been replicated in primate and rodent models of PD. Specifically, MK-801, a non-competitive NMDA antagonist has been demonstrated to relieve parkinsonian symptoms in contralateral limbs when injected unilaterally in MPTP-treated monkeys (Graham et al., 1990), enhanced stepping with the contralateral paw in 6-OHDA-lesioned rats (Kelsey et al., 2004), and potentiates a contralateral circling behavior in 6-OHDA-lesioned rats at 4-fold longer responses than administration of DA alone (St-Pierre and Bedard, 1995). Interestingly, some NMDA receptor antagonists have been shown to potentiate the antiparkinsonian effects of L-Dopa and reduce the motor complications and dyskinesias associated with chronic L-Dopa treatment in both rodent and primate models, suggesting that these drugs may be most useful in combination with L-Dopa therapy (Loschmann et al., 1991; Engber et al., 1994; Marin et al., 1996; Blanchet et al., 1999).

NMDA receptors are known to mediate excitotoxic cell death caused by glutamate, therefore it would be predicted that NMDA antagonists may slow degeneration of nigral neurons. Consistent with this, both intranigral infusion and systemic administration of NMDA receptor antagonists has been shown to protect nigral dopaminergic neurons when administered either prior to or in conjunction with intrastriatal/intranigral injection of 1-methyl-4-phenylpyridinium (MPP^+^) in rats (Turski et al., 1991; Srivastava et al., 1993). Similarly, NMDA receptor antagonists have been shown to protect against cell death, DA depletion, and Parkinsonism induced by systemic administration of MPTP in mice and primates (Zuddas et al., 1992a,b; Lange et al., 1993). These neuroprotective effects were replicated in 6-OHDA-lesioned rats with systemic administration of MK-801 reducing cell death in the SN (Armentero et al., 2006).

The value of NMDA receptor blockade in humans, particularly for LIDs is clear, although most are limited by side effects. Once established, LIDs are difficult to treat. Amongst pharmacological treatment, amantadine has been proven to be clinically effective in a small number of clinical trials, while many others have only shown promise in animal models. A double-blind, placebo-controlled clinical study in advanced PD patients has shown that amantadine reduced dyskinesia severity by approximately 60%, and improved motor fluctuations in L-Dopa treated patients (Verhagen Metman et al., 1998). Such findings have resulted in amantadine becoming the current pharmacological standard treatment for dyskinesia. However, the long-term efficacy of amantadine has been questioned as its benefits may decrease over time, though this may be attributed to the relatively short duration of the clinical trial in which the drug was used (Thomas et al., 2004). A more recent study following PD subjects over the course of 1 year did not show any significant loss of benefit suggesting that amantadine may indeed provide long-term anti-dyskinetic effects (Wolf et al., 2010).

Memantine, another NMDA receptor antagonist that is used widely to treat Alzheimer's disease patients, has been suggested to benefit those with Lewy-body-related dementias, with memantine resulting in greater improvement in cognitive scores than placebo groups (Emre et al., 2010). Furthermore, memantine resulted in improvement in LIDs and on-off timing, with the discontinuation of memantine associated with worsening of dyskinesias and motor fluctuations (Varanese et al., 2010). Interestingly, a recent clinical trial has demonstrated that treatment with memantine was associated with lower axial motor symptoms and dyskinesias scores, but did not improve gait (Moreau et al., 2013).

The widespread expression and critical physiological roles of NMDA receptors raises the concern that global inhibition of NMDA receptors may cause severe adverse side effects such as impaired learning, psychosis, and disruption of motor function (Paoletti and Neyton, 2007). However, recent evidence suggests that targeting specific combinations of NMDA receptor subunits may aid in overcoming this concern. Traxoprodil (CP-101,606), a selective NR2B antagonist, significantly decreased parkinsonian motor symptoms and potentiated L-Dopa responses in MPTP-treated monkeys and reversed catalepsy in haloperidol-treated rats (Steece-Collier et al., 2000). Ifenprodil, another NR2B-selective antagonist, has also been demonstrated to reduce motor symptoms in MPTP-treated primates (Nash et al., 2000). Furthermore, these NR2B antagonists improve the efficacy of L-Dopa and reduce the appearance of LIDs in animal models, suggesting that a combination approach may be of clinical relevance (Blanchet et al., 1999; Steece-Collier et al., 2000). These findings in both animal models and clinical trials demonstrate that NMDA receptor antagonism may provide a significant strategy in the treatment of LIDs. Given their ability to also block excitotoxicity, there is potential for these drugs to also show disease-modifying effects.

#### AMPA receptors

AMPA receptors mediate the vast majority of fast excitatory neurotransmission in the CNS and are important regulators of synaptic plasticity (Dingledine et al., 1999; Derkach et al., 2007; Kessels and Malinow, 2009; Wiltgen et al., 2010; Wright and Vissel, 2012). Given the importance of restoring excitatory balance in BG circuits, it would be reasonable to suggest that blockade of AMPA receptors could offer a potential therapeutic target for treating PD.

Preclinical studies have shown contrasting results, with some studies suggesting that AMPA receptor antagonists in animal models do not offer anti-parkinsonian effects when administered alone (Loschmann et al., 1991, 1992; Wachtel et al., 1992), while others have found anti-parkinsonian effects both alone and in combination with dopaminergic therapies. For example, NBQX has been shown to reverse both reserpine-induced muscle rigidity, but not akinesia, in rats and motor deficits in MPTP-lesioned primates (Klockgether et al., 1991) and potentiate L-Dopa-induced reversal of motor deficits in rats and primates with a nigral lesion (Loschmann et al., 1991, 1992; Wachtel et al., 1992). Studies also indicate that AMPA receptor antagonists reduce LIDs in MPTP-lesioned primates, suggesting blockade of AMPA receptors may reduce motor complications associated with chronic L-Dopa therapy (Konitsiotis et al., 2000; Silverdale et al., 2005).

To date, only a select number of clinical trials have been conducted investigating the effect of AMPA receptor antagonists in PD patients. Perampanel, a non-competitive AMPA receptor antagonist, whilst showing to be well tolerated and safe, failed to improve “wearing off” of motor fluctuations (Eggert et al., 2010) or improve daily “off” time in L-Dopa-treated patients (Lees et al., 2012) and furthermore failed to perform better against entacapone, an active comparator (Rascol et al., 2012). Another AMPA receptor antagonist, talampanel, has been evaluated in a number of Phase II clinical trials to investigate its safety and effects on PD symptoms, including LIDs, however, the results of the trials are not yet available (Clinicaltrials.gov references NCT00036296, NCT00108667, NCT00004576).

Topiramate, a commonly used anticonvulsant drug, falls into a separate class of AMPA receptor antagonists as it has been shown to inhibit both AMPA receptors and the GluK1 subunit of kainate receptors (Gryder and Rogawski, 2003). Topiramate has been shown to reduce LIDs in MPTP-lesioned marmosets without altering the antiparkinsonian action of L-Dopa and reduce L-Dopa-induced AIMs in 6-OHDA-lesioned rats in a dose-dependent manner (Silverdale et al., 2005; Kobylecki et al., 2011). The same animals were also found to have no change in locomotor score but had a moderately reduced rotarod performance at the highest dose. These results suggest that topiramate may act as an anti-dyskinetic drug in addition to its known anticonvulsant properties. In addition, topiramate has a synergistic effect with amantadine, another known anti-dyskinetic drug (see above), with subthreshold doses of both drugs in combination attenuating dyskinesias in 6-OHDA-lesioned rats and the MPTP lesioned marmosets (Kobylecki et al., 2011), suggesting that combination with low-dose amantadine may provide a better reduction of dyskinesias with no adverse motor effects.

As topiramate has a good and well-known side-effect profile through its use as an approved anticonvulsant, its use as a parkinsonian therapy may be able to be expedited to a certain extent. However, topiramate does have well-recognized behavioral side effects that could be a potential problem in PD patients, thus some tolerability studies will be required (Aldenkamp et al., 2000; Kanner et al., 2003). Two Phase II clinical trials were initiated into the anti-dyskinetic effect of topiramate in patients with LIDs, however, both trials have since been terminated due to poor recruitment or cessation of funding (Clinicaltrials.gov reference NCT00794313, NCT00296959). The first published double-blind placebo-controlled trial investigating the effect of topiramate on LIDs demonstrated that topiramate in fact increased dyskinesia severity, contrasting the beneficial effects seen in animal studies (Kobylecki et al., 2014). A more recent Phase II clinical trial, which is recruiting patients, will investigate the anti-dyskinetic ability of topiramate as an adjunct to stable treatment with amantadine (Clinicaltrials.gov reference NCT01789047).

#### Metabotropic glutamate receptors

The mGluRs are comprised of eight subunits (mGluR1-mGluR8) and are subdivided into three groups (I, II, and III) based on receptor structure and physiological activity (Niswender and Conn, 2010). Group I mGluRs are expressed throughout the BG and antagonists of these receptors could be expected to derive antiparkinsonian effects by reducing excitatory drive in overactive BG nuclei. Indeed, several negative allosteric modulators of mGluR5 have been shown to have significant effects in animal models. When administered daily, the mGluR5 negative allosteric modulator MPEP has been shown to reverse akinesia in bilateral 6-OHDA-lesioned rats (Breysse et al., 2003). Furthermore, daily administration of MPEP resulted in an induction of ipsilateral rotations in the unilateral 6-OHDA circling model, however, no effect was seen of MPEP on haloperidol-induced catalepsy (Breysse et al., 2002). Interestingly, a combined administration of MPEP with A_2A_ receptor antagonists reversed akinesia induced by 6-OHDA or reserpine in rodents, suggesting that combining mGluR5 and A_2A_ receptor blockade may be beneficial in the symptomatic treatment of PD (Coccurello et al., 2004; Kachroo et al., 2005).

In addition to a potential symptomatic effect, group I mGluR antagonism has been suggested to alleviate LIDs. Analysis of the putamen and pallidum of dyskinetic MPTP-treated primates showed an increase in mGluR5 binding, which was normalized when dyskinesias were prevented by NMDA receptor blockade (Samadi et al., 2008). Furthermore, the mGluR5 antagonist MTEP was shown to prevent dyskinesias induced by an acute challenge of L-Dopa following weeks of L-Dopa priming in 6-OHDA-lesioned rats (Dekundy et al., 2006; Rylander et al., 2009). More recently, monkeys rendered parkinsonian with MPTP displayed significantly lower dyskinesias when treated with MPEP + L-Dopa compared to those treated with L-Dopa alone (Morin et al., 2013a,b). In contrast, the selective mGluR1 antagonists such as EMQMCM or AIDA were shown to be ineffective in a rodent model of LIDs (Dekundy et al., 2006).

Preclinical studies have also suggested a potential neuroprotective role of mGluRs in the early stage of PD. Repeat intranigral injections of either LY36785, a mGluR1 antagonist or MPEP, a mGluR5 antagonist, have been demonstrated to attenuate the loss of nigral neurons and striatal DA levels in 6-OHDA lesioned rats (Vernon et al., 2005). Furthermore, subchronic intranigral injections with LY36785 or MPEP slowed dopaminergic cell loss in rats that were already undergoing nigrostriatal degeneration by 6-OHDA, suggesting a potential neurorescue effect of group I mGluR antagonism (Vernon et al., 2007). In addition, mGluR5 has been linked to the PD protein alpha-synuclein, with levels of mGluR5 increasing and co-expressing with alpha-synuclein in the BG of alpha-synuclein transgenic mice (Price et al., 2010). Furthermore, these mice displayed an impaired motor performance, which was reversed with MPEP, suggesting that mGluR5 may directly interact with alpha-synuclein, contributing to its activation and role in cell death (Price et al., 2010).

There are a number of clinical trials investigating the effect of small molecules that target mGluRs on various aspects of PD, however, a number of these are either in development or have not published the completed study results. The mGluR5 negative allosteric modulator ADX48621 (dipraglurant), was revealed to be safe and tolerable in three Phase I studies of healthy subjects, allowing for further investigations into its safety, tolerability and efficacy in PD patients with moderate to severe LIDs. In a Phase IIa double-blind, placebo-controlled, multi-center study (Clinicaltrials.gov reference NCT01336088), dipraglurant was administered at increasing doses with L-Dopa for 4 weeks. The results showed no treatment effects on any of the safety monitoring variables and adverse events were common in both treatment groups. Furthermore, dipraglurant reduced dyskinesia severity, with no increase in “off” time and a greater increase in “on” time without dyskinesia. Interestingly, dipraglurant was as effective in those in the study that had undergone deep brain stimulation (DBS) as in non-DBS subjects (MichaelJFoxFoundation, 2012).

AFQ056 is another potent, subtype selective inhibitor of mGluR5 that has been investigated in clinical trials. A Phase II study investigated multiple oral dose titration of AFQ056 in PD patients to assess its safety, tolerability and efficacy in reducing LIDs over a period of 16 days. AFQ056 was shown to significantly reduce Lang-Fahn Activities of Daily Living Dyskinesia Scale (LFADLDS) scores on day 16 and significantly reduce average AIMs on days 12 and 16 (Berg et al., 2011). Based on these results an open-label Phase II study is currently ongoing to determine the long-term safety, tolerability and efficacy of AFQ056 in those patients who were eligible for, participated in, and completed the previous study (Clinicaltrials.gov reference number NCT01173731). A recently completed Phase IIb double-blind, placebo-controlled study investigating the effect of AFQ056 on LIDs in patients with moderate to severe PD demonstrated significant improvements in dyskinesia severity at the highest dose of AFQ056 administered. However, for all other doses evaluated the primary endpoint was not met, despite a dose-dependent efficacy (Stocchi et al., 2013). An additional Phase II study is currently underway to investigate the efficacy and safety of modified release AFQ056, with or without the administration of amantadine, in patients with LIDs (Clinicaltrials.gov reference number NCT01491529).

### Serotonin receptor agonists

In the normal brain, there is a dense serotonergic innervation of the BG from the raphe nuclei, with the striatum, globus pallidus, and output nuclei receiving high levels of input (Lavoie and Parent, 1990). Postmortem studies in PD patients have reported conflicting results with respect to serotonin (5-HT) markers, however, there is a general consensus that 5-HT is decreased in the PD brain, suggesting 5-HT may play a potential role in the disease (Kish, 2003; Scholtissen et al., 2006). Compared to the changes seen in human tissue, the alteration of the 5-HT system differs in animal models of PD, with differences seen depending on toxin, species of animal that is used, and brain region examined (Erinoff and Snodgrass, 1986; Hara et al., 1987; Zhou et al., 1991; Rousselet et al., 2003; Boulet et al., 2008).

Regardless, recent evidence strongly suggests that 5-HT neurons may play an important role in L-Dopa-induced DA release and thus LIDs. It has been suggested that this effect is due to released DA from spared 5-HT fiber terminals subsequent to their uptake of repetitive, low doses of L-Dopa. It is thought that, since these terminals express both aromatic l-amino acid decarboxylase (AADC) and vesicular monoamine transporter 2 (VMAT2), the 5-HT fiber terminals are able to take up L-Dopa, convert it into DA, store it within the neuron and then release it in the DA-depleted brain (Arai et al., 1995; Carta and Bezard, 2011). The 5-HT terminals therefore facilitate the therapeutic action of L-Dopa but the raphe-terminals lack auto-regulatory feedback mechanisms for DA release and can result in excessively enhanced DA levels in the extracellular space (Carta et al., 2007; Lindgren et al., 2010). This fluctuation in DA levels then leads to supersensitive responses of striatal neurons and can trigger dyskinesias. A study conducted by Carta et al. (2007) demonstrated that lesioning 5-HT afferents to the striatum with 5,7-DHT, or inhibiting neurotransmitter release from 5-HT terminals by administering 5-HT agonists, eradicated established LIDs in animals with both a partial and complete dopaminergic lesion (Carta et al., 2007). Furthermore, L-Dopa-naïve, non-dyskinetic rats that had a complete removal of 5-HT afferents failed to develop LIDs when L-Dopa was subsequently administered.

This finding that 5-HT may regulate L-Dopa-induced DA release has led to the investigation of molecules acting on 5-HT receptors as anti-dyskinetic drugs. To date, there are 14 known distinct subtypes of the 5-HT receptor with many more isoforms. Nevertheless, the autoreceptors 5-HT_1A_ and 5-HT_1B_ are among the most studied in PD (Peroutka, 1995). Stimulating 5-HT_1A_ and 5-HT_1B_ via 8-OH-DPAT and CP-94253, respectively, has been shown to reduce the activity of the raphe-striatal neurons, blunt the extracellular DA release in the striatum and attenuate the expression of LIDs in rats lesioned with 6-OHDA (Carta et al., 2007; Lindgren et al., 2010). These findings have been replicated in non-human primate models of PD with dyskinetic monkeys receiving a combination of 5-HT_1A_ and 5-HT_1B_ agonists displaying an 80% reduction of dyskinesias without a significant worsening of their parkinsonian scores compared to L-Dopa-only treated animals (Munoz et al., 2008).

The findings in animal models have resulted in an open-label, multicenter trial of the 5-HT_1A_ agonist sarizotan's safety, tolerability and efficacy in patients with advanced PD complicated by troublesome LIDs. Sarizotan treatment significantly reduced dyskinesias as measured by home diary, AIMS, and UPDRS scores. Furthermore, dyskinesia benefits were obtained without change in total “off” time or in change from baseline mean UPDRS scores. However, while initially exciting, several patients experienced worsening parkinsonism with sarizotan or with increasing doses, due to the fact that sarizotan not only antagonizes 5-HT_1A_ receptors but also blocks the D4 DA receptor (Olanow et al., 2004). These disappointing results do not rule out the value of targeting 5-HT receptors, but instead suggest that sarizotan may not be the ideal therapeutic for treating LIDs.

These anti-dyskinetic effects were replicated in one double-blind, placebo-controlled study of sarizotan (Bara-Jimenez et al., 2005) but were in contrast to a more recent study using a larger patient population in which no significant changes were found in dyskinesias measures but an improvement in UPDRS and “off” time was revealed (Goetz et al., 2007). This lack of efficacy of sariztoan for LIDs could be attributed to the low dose given during this trial. A number of small clinical trials have been conducted investigating other 5-HT_1A_ receptor agonists. In a double-blind, placebo-controlled study, buspirone significantly lessened the severity of LIDs in 5 out of the 7 patients but proved ineffective in the remaining 2 who had the mildest dyskinesias (Bonifati et al., 1994). Another 5-HT_1A_ agonist with 5-HT_2_ antagonism, mirtazapine, was shown in an open label study to be moderately effective in reducing LIDs both alone and in combination with amantadine (Meco et al., 2003). In addition a number of partial 5-HT agonists, including clozapine (Durif et al., 1997, 2004), tandospirone (Kannari et al., 2002) and aripiprazole (Meco et al., 2009) have also shown some limited success at attenuating dyskinesias.

Clinical trials of 5-HT receptor agonists as anti-dyskinetic agents have been somewhat disappointing, though it should be noted many of these trials consisted of small patient populations, thus drawing meaningful conclusions from their results is difficult. Furthermore, these studies have focused on individual autoreceptor agonism, rather than a dual 5-HT_1A_ and 5-HT_1B_ agonism approach. This raises the possibility that the failure of clinical trials to date, at least those targeting 5-HT receptors, reflects a failure to accurately take the lessons learned in animal models to the clinical trials, namely that dual 5-HT_1A_ and 5-HT_1B_ agonism may be required. A recent study demonstrating the antidyskinetic effect of eltoprazine, a mixed 5-HT_1A_/5-HT_1B_ receptor agonist, in both rodents and non-human primates shows promise that progress is being made in this treatment strategy (Bezard et al., 2013).

### Adrenergic receptor antagonists

In addition to the well-documented loss of SN DA cells, it has been shown that norepinephrine (NE) neurons of the locus coeruleus also undergo degeneration in PD and may even precede the death of DA neurons (Zarow et al., 2003; Fornai et al., 2007; Mcmillan et al., 2011). These adrenergic neurons originating from the LC appear to play a protective role by establishing the extent of nigral degeneration induced by both neurotoxic damage and by pathological events underlying PD (Mavridis et al., 1991; Fornai et al., 2007; Rommelfanger et al., 2007). It is therefore thought that activation of adrenergic pathways through the blockade of adrenergic autoreceptors, in particular the α_2_ receptor, should oppose the nigrostriatal dopaminergic degeneration and the subsequent motor deficits in PD.

It has been suggested that activation of α_2_ adrenergic receptors can facilitate movements produced by the activation of the direct pathway of the BG, thus highlighting enhanced α_2_ receptor stimulation as a potential mechanism underlying LIDs (Hill and Brotchie, 1999). Indeed, the α_2_ adrenergic receptor antagonist yohimbine significantly reduced L-Dopa-induced hyperkinesia in 6-OHDA-lesioned rats. These effects did not reflect non-specific reductions in locomotion, as the rats did not display significantly reduced levels of spontaneous locomotion, thus indicating a specific effect on L-Dopa-induced effects (Henry et al., 1998). Furthermore, rats receiving 6-OHDA without concomitant administration of desipramine, thus lesioning both the DA and NE systems, demonstrated markedly reduced L-Dopa-induced rotations (Barnum et al., 2012). In addition, the α_2_ adrenergic receptor antagonist idazoxan was effective in alleviating the expression of AIMs in rats lesioned with 6-OHDA alone (Barnum et al., 2012) and reducing haloperidol-induced catalepsy (Invernizzi et al., 2003). These results were confirmed in studies of MPTP-lesioned non-human primates with α_2_ adrenergic receptor antagonists significantly reducing LIDs without compromising the anti-parkinsonian action of L-Dopa (Gomez-Mancilla and Bedard, 1993; Henry et al., 1999; Fox et al., 2001; Savola et al., 2003). Fipamezole, a more recently developed α_2_ adrenergic receptor antagonist, has also been shown to extend both the duration and quality of L-Dopa action with total “on” time increased by up 75% and “on time without disabling dyskinesia” increased by up to 98% in MPTP-lesioned macaques (Johnston et al., 2010).

To our knowledge, only idazoxan and fipamezole have subsequently progressed to clinical trial investigations. In a randomized, placebo-controlled pilot study, the effects of single oral doses of idazoxan on motor disability and LIDs following an acute oral challenge of L-Dopa was assessed in 18 patients with PD, with idazoxan able to improve the severity of LIDs without a concomitant deterioration of the antiparkinsonian effect of L-Dopa (Rascol et al., 2001). Meanwhile a recent 28 day dose-escalating effect of fipamezole was investigated in a Phase II double-blind, randomized, placebo-controlled study in PD patients experiencing LIDs. The total study population showed no statistically significant primary endpoint difference between fipamezole and placebo, however, this may be attributed to the non-homogenous US and Indian study populations. Therefore analysis of a subgroup of US subjects was conducted with fipamezole reducing LIDs in a dose-dependent manner and inducing mild, transient blood pressure elevation, which was considered as an acceptable profile of adverse events (Lewitt et al., 2012). There have been 3 other clinical trials investigating fipamezole that have been completed, however, no study results have been published (Clinicaltrials.gov references NCT01149811, NCT01140841, NCT00040209).

### Calcium channel blockers

One of the most popular held theories of aging is that it is a direct consequence of accumulated mitochondrial DNA damage produced by reactive oxygen species (ROS) and free radicals generated in the course of oxidative phosphorylation (Harman, 1972). It could be hypothesized that the reliance of DA neurons on the metabolically expensive action of sequestering calcium (Ca^2+^), results in these neurons aging more rapidly than other types of neurons. Indeed, histological estimates of normal aging-related cell death suggest this may be the case (Stark and Pakkenberg, 2004).

It has been suggested that the specific physiology of nigral DA neurons may provide some answers as to why there is preferential loss of these cells in PD. Unlike most other neurons in the CNS, the DA neurons of the SN generate rhythmic action potentials in the absence of synaptic input. Furthermore, most other neurons use channels that allow sodium (Na^+^) ions across the membrane to mediate pacemaking whereas nigral DA neurons rely upon L-type Ca^2+^ channels (Bonci et al., 1998). These L-type channels have a pore-forming Cav1.3 subunit rather than the cardiac Cav1.2 subunit (Striessnig et al., 2006), however, as Ca^2+^ is central to a wide variety of cellular processes this reliance on Cav1.3 Ca^2+^ channels may be problematic. In addition, in nigral DA neurons the Ca^2+^ channels are open much of the time, thus the magnitude of Ca^2+^ influx is greater and subsequently increases the burden and vulnerability of the cell to failed Ca^2+^ homeostasis leading to cell death (Wilson and Callaway, 2000).

Interestingly, the reliance of nigral DA neurons upon Ca^2+^ channels to drive pacemaking is developmentally regulated. Young neurons generate their activity autonomously via Na^+^ channels, with this mechanism retained in adult neurons but in a latent capacity (Chan et al., 2010). Furthermore, sustained block of Cav1.3 Ca^2+^ channel-induced pacemaking “rejuvenates” this juvenile mechanism and results in DA neurons spiking at normal rates and mice displaying no obvious motor, learning or cognitive deficits (Chan et al., 2007). If PD is a consequence of the accelerated aging of neurons that rely heavily upon Ca^2+^ channels, then reducing this dependence and forcing back to an L-type channel independent mechanism should slow PD progression and/or delay clinical manifestations of PD.

Ca^2+^ channel blockers have been used for decades to treat hypertension. *Post-hoc* analysis of patients treated with one of the hypertensive drug classes known as dihydropyridines, revealed a lower incidence of PD (Rodnitzky, 1999). This positive effect resulted in numerous investigations of this class of drugs, in particular isradipine, in animal models of PD. In one study, mice systemically administered isradipine with slow-release, subcutaneous pellets showed a strong protection of nigral neurons against 6-OHDA-induced cell death (Chan et al., 2010). This neuroprotection was confirmed using an osmotic pump delivery system, with isradipine providing a dose-dependent sparing of DA fibers in the striatum and nigral DA neurons (Ilijic et al., 2011). Isradipine was also shown to protect against MPTP-induced toxicity in mice with a reduction of loss of dopaminergic SN cells by nearly half whilst preventing the development of motor deficits (Chan et al., 2007). Interestingly, subcutaneous isradipine administration in rats lesioned with 6-OHDA attenuated L-Dopa-induced rotational behavior and AIMs in a dose-dependent manner, suggesting that blocking L-type Ca^2+^ channels may also provide a symptomatic benefit in addition to its neuroprotective role (Schuster et al., 2009).

While there is a significant benefit of isradipine in animal models of PD, a potential therapeutic caveat of using 1,4-dihydropyridines (DHPs), a group of drugs of which isradipine belongs to, is that they are not selective. Among the DHPs, isradipine has the highest affinity for Cav1.3 channels, however, it is still Cav1.2 selective (Lipscombe et al., 2004). A recent study aimed to overcome this issue by performing high-throughput screening of chemical libraries with subsequent modification, identifying 1-(3-chlorophenethyl)-3-cyclopentylpyrimidine-2,4,6-(1H,3H,5H)-trione as a potent and highly selective Cav1.3 L-type Ca^2+^ channel antagonist (Kang et al., 2012). It is hopeful that this study will open the field to further development of selective antagonists and subsequent preclinical and clinical evaluation.

There is currently very little clinical evidence of the use of Ca^2+^ channel blockers in patients with PD. One clinical trial, which followed on from an earlier safety and tolerability study, showed that isradipine was reasonably tolerated. Furthermore the main side effect of leg oedema was reversed with dose reduction (Simuni et al., 2010; Rees et al., 2011). This was confirmed in a more recent study demonstrating a dose-dependent tolerability of isradipine, however, no differences in reduction of symptoms were found (Parkinson Study Group, 2013). However, the authors note that this may be due to their design not being powered for efficacy or futility analysis. However, the identification of a tolerable dose supports the use of isradipine for future efficacy trials. Importantly a large Phase III clinical trial of isradipine is now in development (Michael J Fox Foundation, 2014).

### Glucagon-like peptide 1 (GLP-1) agonist

The apparent link between diabetes and Parkinson's risk has gained significant attention in recent times and it is increasingly suggested, including by us, that anti-diabetes drugs may offer benefit for neurological diseases, independent of their anti-diabetic actions (Clark et al., 2012; Clark and Vissel, 2013). The insulin-tropic hormone glucagon-like peptide-1 (GLP-1) is an endogenous peptide that has been developed to treat diabetes and that controls plasma glucose levels. Recently, GLP-1 and analogs with extended action have emerged as a highly novel target to treat PD.

Exendin-4, a long-acting analog of GLP-1, was shown to have neurotrophic and neuroprotective properties, similar to other neurotrophic factors, in cultured PC12 cells subjected to excitotoxic stress (Perry et al., 2002a,b), suggesting that stimulation of GLP-1 receptors may be therapeutically beneficial in neurodegenerative disorders such as PD. We have extensively reviewed the mechanisms by which GLP-1 agonists may show benefit for neurodegenerative disease (Clark et al., 2012; Clark and Vissel, 2013).

Exendin-4 was found to protect ventral dopaminergic cells in culture that were exposed to 6-OHDA, an effect that was replicated in SH-SY5Y cells, which can be differentiated into neurons with a dopaminergic phenotype (Li et al., 2009). This same study revealed that exendin-4 treatment protected against MPTP-induced toxicity, with mice receiving exendin-4 having significantly higher numbers of dopaminergic neurons, preserved DA levels and improved scores in multiple motor testing paradigms.

Exendin-4 may also be able to arrest and possibly reverse nigrostriatal lesions once the neurodegeneration has already begun. Mice receiving exendin-4 1 week following 6-OHDA-lesioning displayed significantly lower apomorphine-induced circling, higher striatal DA concentrations and nigral tyrosine hydroxylase (TH) staining, indicating a potential neurorescue effect (Harkavyi et al., 2008). Interestingly, these protective effects were also found when mice received SN lesions of lipopolysaccharide (LPS) instead of 6-OHDA, suggesting that exendin-4 may also have anti-inflammatory effects.

Clinically, exendin-4 has been used as a treatment for type 2 diabetes since 2005, with very little investigation of its use in PD. However, a recent Phase II clinical trial aimed to show proof of concept of exendin-4 on the progress of 45 patients with moderate PD. In the single-blind trial, patients received subcutaneous injection of exendin-4 for 12 months and showed improved motor and cognitive function that persisted for 2 months after treatment had stopped (Aviles-Olmos et al., 2013). A potential caveat to this study was the lack of placebo control. However, the authors note this was due to the complex drug device and the cost of manufacture of a matched placebo. Nevertheless, this cost-efficient proof-of-concept study may open doors for future rigorous double-blind, placebo-controlled trials.

### Iron chelators

A healthy SN has a higher concentration of iron than the liver, which is the main store of iron in the body (Mastroberardino et al., 2009). Iron in neuronal cells is usually bound to ferritin, neuromelanin, or stored in the lysosome and iron homeostasis is crucial for multiple brain functions and is tightly regulated by a number of mechanisms (Li et al., 2011). It has become apparent that iron mismanagement within the brain may contribute to a variety of neurological disorders, including PD. Iron accumulation in the SN of PD patients was first described in 1924, interestingly before the identification of DA deficiency, and has been suggested as a contributing factor for DA neuron degeneration (Berg and Hochstrasser, 2006). It has been suggested that an increase in iron results in oxidative stress within the SN and exacerbates the neurotoxicity of other alleged pathogens, such as neuromelanin and endogenous DA, and consequently lead to preferential DA neuron degeneration (Cozzi et al., 2010).

As 6-OHDA is thought to induce nigrostriatal degeneration via metal-catalyzed free radical formation, the effect of iron chelators was first investigated in this model. Intracerebroventricular administration of desferrioxamine (also known as desferoxamine, DFO, or desferal) prior to 6-OHDA has been demonstrated to significantly protect against reduction in striatal DA content and a normalization of DA release in rats. Desferrioxamine-pretreated rats also exhibited normal behavioral responses in contrast to animals treated with 6-OHDA alone, which demonstrated significantly impaired rearing and spontaneous movements in a novel environment (Ben-Shachar et al., 1991, 1992; Youdim et al., 2004). Furthermore, desferrioxamine alone did not affect striatal TH activity or DA metabolism. These results led to the development of the novel brain-permeable iron chelator, VK-28, with pretreatment able to completely protect against 6-OHDA-induced depletion of DA and its metabolites in rats (Shachar et al., 2004). This neuroprotective effect has been replicated in the MPTP model, with the iron chelators desferrioxamine, clioquinol, and M30 significantly increasing DA levels and nigral dopamine neuron numbers following MPTP administration (Lan and Jiang, 1997; Kaur et al., 2003; Gal et al., 2005; Youdim, 2012).

Iron chelators are already used in the clinic as treatment strategies for various conditions. Desferrioxamine has been the most widely used iron chelator over the last three decades, however, its inability to cross the blood brain barrier in concentrations that are therapeutically efficacious has restricted its use in neurodegenerative disorders. The chelators deferiprone and clioquinal are able to be administered orally and thus have a great advantage over desferrioxamine. A number of Phase II clinical trials investigating the safety and efficacy of deferiprone in patients with PD, with subjects assessed via UPDRS scores and, when possible, by magnetic resonance imaging are currently underway (Clinicaltrials.gov references NCT01539837, NCT00907283). A recently completed trial in early PD patients demonstrated a significant reduction in accumulated iron levels in the SN and a corresponding improvement in motor symptoms in patients receiving immediate treatment of deferiprone, while those assigned to a delayed start paradigm experienced worsening of symptoms initially, followed by improvement in motor function upon treatment with deferiprone (Devos et al., 2014). Further multi-center trials will be required to consolidate these findings and investigate the mechanism of action of deferiprone in the clinical setting.

### Anti-inflammatories

The term “neuro-inflammation” is currently ill defined. However, for now, as is apparent from the literature, inflammation in the brain will generally continue to be defined by the presence of reactive astrocytes and amoeboid microglia, together with expression of inflammatory cytokines. It is widely accepted that such inflammation occurs in the PD brain, although it is currently unknown as to what initiates the inflammatory response and whether it is part of an attempt, at least initially, to repair the brain or a consequence of nigrostriatal degeneration. We, and others have suggested that the issue may not be inflammation in the CNS *per se*, but may be, more importantly, an interference with normal glial function at the synapse (Abdipranoto-Cowley et al., 2009; Clark et al., 2010; Morris et al., 2013). If true, then the therapeutic target for PD and neurodegenerative disease more generally will not ultimately be inflammation, but rather will be focused on restoring synapse function, in part through restoring the normal functions of glia at the synapse.

The first evidence for a role of inflammation in PD resulted from a study conducted on post mortem brains, which demonstrated reactive microglia in the SN in patients with PD (Mcgeer et al., 1988). Reactive astrocytes are generally thought to be absent or described as mild or moderate in PD brains (Mirza et al., 2000). Ever since, there have been a large number of studies that have supported the role of activated microglia and increased levels of inflammatory mediators such as cytokines, chemokines and ROS in the pathology of PD (Mogi et al., 1994; Banati et al., 1998; Knott et al., 2000; Reale et al., 2009). Whilst it has been suggested that mild activation of microglia has beneficial effects, chronic activation, as is evident in PD, is thought to contribute to the death of otherwise viable cells (Gao and Hong, 2008). Furthermore, there is a wealth of evidence from animal models regarding the role of inflammation in the pathogenesis of PD, with inflammatory markers identified in 6-OHDA (Marinova-Mutafchieva et al., 2009; Harms et al., 2010; Wachter et al., 2011), MPTP (Grunblatt et al., 2001; Mcgeer et al., 2003; Yasuda et al., 2008; Barcia et al., 2011), paraquat (Cicchetti et al., 2005; Mitra et al., 2011) and rotenone models of PD (Sherer et al., 2003; Phinney et al., 2006).

Given the observation that inflammation is seen in the PD brain, there has been interest in the potential of anti-inflammatory drugs for treating PD. Non-steroidal anti-inflammatory drugs (NSAIDs) are the main drugs used to reduce the effects of inflammation. NSAIDs work by inhibiting cyclooxygenase (COX), an enzyme that catalyzes the formation of prostaglandins, as well as having an inhibitory effect in the synthesis of nitric oxide radical (Asanuma and Miyazaki, 2007). A number of anti-inflammatories have been investigated in animal models of PD including aspirin and its metabolite salicyclic acid, as well as COX-1 and COX-2 inhibitors.

Both aspirin and salicyclic acid have been shown to be neuroprotective against MPTP-induced striatal DA depletion and dopaminergic nigral death in mice through effective scavenging of hydroxyl radicals (Aubin et al., 1998; Ferger et al., 1999). However, subsequent studies indicated that COX-1 and COX-2 enzymes may play a bigger role. This was confirmed in studies investigating the COX inhibitor meloxicam, with high doses showing almost complete protection against MPTP-induced toxicity with decreased striatal DA depletion, attenuation of reduction of TH-immunoreactive cells in the SN and MPTP-induced decrease in locomotor activity (Teismann and Ferger, 2001). Another COX inhibitor, indomethacin, was also shown to protect dopaminergic neurons within the SN in mice following MPTP and also decreased microglial activation and lymphocytic infiltration in the damaged areas, however, it appeared to be toxic at high doses (Kurkowska-Jastrzebska et al., 2002). COX inhibitors have also shown to be protective against the toxic effects of 6-OHDA. Celocoxib, a COX-2 inhibitor, when administered to rats lesioned with 6-OHDA protected against intrastriatal degeneration and resulted in decreased microglial activation in the striatum and ventral midbrain (Sanchez-Pernaute et al., 2004).

Despite the evidence of inflammation in postmortem brains and various animal models, the use of NSAIDs has not been formally tested in clinical trials of PD. However, epidemiological studies suggest that the use of non-aspirin NSAIDs is associated with a 15% reduction in risk of PD, a 29% reduction with regular use and a 21% reduction with long-term use, suggestive of a dose-response relationship (Gagne and Power, 2010). However, many questions remain before their use as a PD therapy, such as the most appropriate patient population, drug type, dose, and length of administration. Furthermore, the use of NSAIDs has been associated with adverse effects such as gastrointestinal side effects, which may hamper their use at sufficient doses in the clinic.

The side effects of NSAIDs limit the dose at which they can be used in the clinic. Consequently, it is possible that the limited evidence supporting the efficacy of anti-inflammatories may result from a problem as simple as a lack of sufficient dosing. Indeed high doses of anti-inflammatories have been used in animal models to suppress inflammation (Abdipranoto-Cowley et al., 2009; Mertens et al., 2013), doses that would cause substantial side effects with chronic use in humans.

The anti-inflammatory properties of minocycline provide an alternative to the use of NSAIDs in the clinic. As one of the treatment arms in the Phase II randomized, double-blind NET-PD clinical trial, which also investigated creatine, twice daily administration of minocycline was examined for its potential to alter the course of early PD relative to a predetermined futility threshold of 30% reduction in UPDRS progression. The results demonstrated that minocycline could not be rejected as futile for future studies when examined against this predetermined threshold (The Ninds Net-Pd Investigators, 2006). An additional 6-month follow up study demonstrated that by 18 months from starting minocycline, 62% of patients required further symptomatic treatment. However, minocycline did not adversely affect this symptomatic treatment or increase adverse events (The Ninds Net-Pd Investigators, 2008).

There continues to be an ever-increasing literature on the role of inflammation in PD (Amor et al., 2010; Hirsch et al., 2012; Phani et al., 2012), however, understanding of neuroinflammation is still in its earliest stages and this may be constraining advances in this area. More generally, a great deal remains to be learnt regarding the role of glial cells and the mechanisms that regulate their function in the normal and diseased brain (Morris et al., 2013). It is conceivable that the issue is not classical inflammation at all, but rather a failure of normal glial function occurring in the diseased brain. Thus, general anti-inflammatory approaches may not show the specific effects needed for disease-modification and more specific targeting of signaling mechanisms that regulate glial function and responses will ultimately be required.

### Gene therapy

A great advance in the field of PD therapeutics and an alternative to traditional pharmacological approaches includes the viral vector-mediated targeted delivery of therapeutic genes such as aromatic amino acid decarboxylase (AADC) and glutamic acid-decarboxylase (GAD). The advantage of gene therapy lies in the potential to deliver therapies in a tightly controlled manner to specific brain regions, limiting the chance of off-target effects. The disadvantages continue to include the inability to tightly regulate the amount delivered and the fact that the treatments are largely irreversible. For these reasons, while promising, gene therapy has been regarded as experimental.

#### Aromatic L-amino acid decarboxylase (AADC)

Aromatic L-amino acid decarboxylase (AADC) is an enzyme that converts L-Dopa to DA, offering an attractive target to endogenously stimulate production of DA in surviving neurons. Using the adeno-associated virus (AAV) as a delivery method, AADC has been shown to decarboxylate endogenous levels of L-Dopa more efficiently when injected into animal models of PD. In rats lesioned with 6-OHDA, animals that received AAV-AADC demonstrated rotational scores that were strongly correlated with AADC activity in the lesioned striatum and restoration of DA production to 50% of normal levels by 12 weeks after receiving the gene therapy (Sanchez-Pernaute et al., 2001). In addition, the gene transfer-induced increase in striatal decarboxylation of peripherally administered L-Dopa was shown to remain undiminished over a 6 month period and expression of the transgene was detected for at least 1 year (Leff et al., 1999). Furthermore, simultaneous infection of 6-OHDA-lesioned rats with AAV-TH and AAV-AADC resulted in more effective DA production and a greater behavioral recovery than was seen in rats receiving AAV-TH alone (Fan et al., 1998).

In two Phase I clinical trials, 6-month evaluation of AAV-AADC into the striatum improved off-medication motor function of almost 50% of UPDRS scores and increased AADC activity in the striatum, however, three out of the 10 subjects in one of the studies suffered hemorrhage along the trajectory of the injecting catheter (Christine et al., 2009; Muramatsu et al., 2010). Clearly such risks are unacceptable and will need to be addressed. Nevertheless, a long-term follow up study revealed that AADC gene expression was maintained at least 4 years after administration, indicating transgene stability (Mittermeyer et al., 2014) though further trials will be needed to determine if a higher vector dose is able to induce a similar stable effect on motor symptoms.

A recently completed Phase I/II clinical trial investigating ProSavin, a lentiviral vector encoding AADC, along with tyrosine hydroxylase and cyclohydrolase 1, demonstrated a significant improvement in UPDRS scores up to 12 months following ProSavin administration (Palfi et al., 2014). While these positive outcomes describe the first use of a lentiviral-based vector in a neurodegenerative disease, further optimization of mode and dose of delivery will be required before proceeding to further clinical trials.

#### Glutamic acid decarboxylase (GAD)

As noted above it is thought that nigrostriatal degeneration leads to excessive inhibitory output from the GPi and SNr. This is due to disinhibition of the STN, which drives the GPi and SNr via the release of glutamate, suggesting that a gene transfer strategy that enhances GABA transmission in the STN and its terminal regions via GAD may be therapeutically beneficial. A study conducted by Luo et al. (2002) showed that GAD65 into the STN mediated an increase in survival of midbrain dopaminergic neurons. Furthermore, rotation rates in GAD65-transduced 6-OHDA-lesioned rats were decreased by 65%, a result confirmed in subsequent studies (Luo et al., 2002; Lee et al., 2005). In addition, administration of AAV-GAD65 into the STN revealed significant improvement in bradykinesia, gross motor skills, and tremor in macaques rendered hemiparkinsonian by MPTP (Emborg et al., 2007).

Following these promising animal studies, the administration of AAV-GAD was examined in PD patients. An open-label study in 12 patients with advanced PD followed over a 12-month period after unilateral injections of AAV-GAD into the STN, demonstrated a significant improvement in UPDRS scores, expressed predominantly on the side of the body contralateral to the surgery and were seen after 3 months and persisted up to 12 months after gene therapy (Kaplitt et al., 2007). A phase II sham-surgery controlled study of bilateral infusions of AAV-GAD into the STN of progressive L-Dopa responsive PD patients showed significant reductions in off-medication UPDRS motor scores. With no serious safety concerns reported so far (Lewitt et al., 2011), this remains as a promising direction of research.

#### Gene therapy for delivery of neurotrophic factors

Viral mediated delivery of the neurotrophic factors glial derived neurotrophic factor (GDNF) and neurturin (NTN) to specific CNS regions have also been extensively explored as potential therapeutics for PD. We will elaborate on these efforts as part of a more general discussion about neurotrophic factors below.

### Neurotrophic factors

While their mechanism of neuroprotective action is poorly understood, there has been a long held interest in the potential benefits of neurotrophic factors for treating PD. Several studies have provided evidence that neurotrophic factors can provide beneficial effects on dopaminergic neurons (Collier and Sortwell, 1999; Rosenblad et al., 1999). Consequently, they have been extensively investigated as described below.

#### Glial derived neurotrophic factor

GDNF is an important survival factor for midbrain dopaminergic neurons and stimulates the growth of process from immature neurons (Lin et al., 1993) providing an attractive therapeutic target for halting PD degeneration. Both the density of TH immunoreactivity and DA levels were significantly rescued in mice in which GDNF was injected into the SN before MPTP lesioning or into the striatum 7 and 16 days after lesioning, indicating both a protective and restorative effect on dopaminergic neurons (Tomac et al., 1995). Administration of GDNF to the region just above the SN 1 week following 6-OHDA lesion resulted in a partial but substantial protection of nigral neurons, but with the remaining neurons appearing significantly atrophied, indicating that proximity of GDNF administration to the site of lesion is important for preserving neural function (Sauer et al., 1995). Indeed, the site of GDNF administration has been shown to have differential effects in rodent models of PD. In rats lesioned with 6-OHDA, prior injection of GDNF into the striatum resulted in preservation of striatal terminals, nigral cell bodies and preservation of motor function while intranigral injections resulted in protection of nigral cell bodies but no subsequent preservation of DA axons or behavioral outcomes (Kirik et al., 2000a).

The beneficial effects of GDNF have also been demonstrated in non-human primate models of PD. Intracerebroventricular GDNF delivered monthly in MPTP-treated monkeys resulted in decreased parkinsonism in a dose responsive manner, attenuated LIDs (Miyoshi et al., 1997; Zhang et al., 1997), and is correlated with increased levels of the DA metabolites 3,4-Dihyroxyphenylacetic acid (DOPAC) and homovanillic acid (HVA) (Gerhardt et al., 1999).

Based on these results, a randomized double-blind clinical trial was initiated using intracebroventricular GDNF infusion. However, the results were disappointing, with no clinical improvement, an absence of neuroprotection and GDNF diffusion in the brain parenchyma (Kordower et al., 1999). Furthermore, a Phase II double-blind, placebo-controlled study conducted by AMGEN of continuous infusion of GDNF into the putamen, while showing the therapy to be well-tolerated did not meet the primary endpoint at 6 months, with no improvement in UPDRS scores. In addition, AMGEN suddenly halted the study as a result of identification of cerebellar lesions in preclinical non-human primate studies, prompting fears for the long-term safety of patients in the trial (Amgen, 2005). Continuous infusion using a minipump system was then trialed, however, these studies provided conflicting results, strengthening gene therapy as a potential alternative delivery method (Nutt et al., 2003; Slevin et al., 2007).

Several studies have been conducted in animal models of PD using adenovirus, lentivirus and AAV-based vectors to express GDNF in the striatum or SN. One study, using a lentiviral vector, resulted in an eightfold increase in TH immunoreactive neurons in aged monkeys and a sevenfold increase in those rendered parkinsonian with MPTP (Palfi et al., 2002). However, the most promising studies are those utilizing the AAV vector, with GDNF expression achieved for up to 6 months in rats with a single injection of AAV-GDNF. Furthermore, AAV-GDNF administered to both the nigra and striatum was revealed to be capable of providing complete protection of nigral neurons following 6-OHDA. However, as occurred with infused GDNF, the site of GDNF delivery was crucial to functional outcome with only AAV-GDNF administered to the striatum providing functional recovery (Kirik et al., 2000b). Another study, using an AAV vector revealed that a delayed delivery of GDNF in 6-OHDA lesioned rats resulted in significantly higher density of TH immunoreactive fibers in the striatum and neurons in the SN as well as higher levels of DA and its metabolites, resulting in a significant behavioral recovery (Wang et al., 2002).

These beneficial effects have been replicated in non-human primate models of PD with AAV-GDNF shown to protect nigral neurons, provide a partial protection of DA fibers in the striatum, and bring about a clinical improvement in monkeys lesioned with 6-OHDA and MPTP (Eslamboli et al., 2003; Eberling et al., 2009; Johnston et al., 2009). Despite the successful outcomes in animal models of PD, clinical trials assessing the efficacy of AAV-GDNF treatment have yet to be conducted, with only one Phase I trial utilizing convection enhanced delivery of AAV-GDNF currently recruiting participants (ClinicalTrials.gov NCT01621581).

#### Neurturin

Another neurotrophic factor that is a close homolog of GDNF, called neurturin (NTN), emerged as a potential therapeutic target. NTN is 40% identical to GDNF and while it is known to support the survival of a variety of peripheral neurons, the complete extent of its function is yet to be determined (Kotzbauer et al., 1996). Much like GDNF, the injection of recombinant NTN has demonstrated variable results in animal models of PD. In one study, the delivery of NTN into the striatum after 6-OHDA lesioning in rats resulted in a 72% protection of nigral DA neurons but failed to rescue DA levels in the striatum (Rosenblad et al., 1999). In contrast, a study by Oiwa et al. (2002) demonstrated that NTN administered 12 weeks following 6-OHDA failed to protect nigral neurons but increased striatal DA fibers (Oiwa et al., 2002).

Again, the use of viral vector-mediated delivery has provided the most efficacious method for the therapeutic administration of NTN. A recombinant AAV2-based vector encoding for human NTN, known as CERE-120, has been developed by Ceregene Inc. When administered into the striatum in rats, NTN expression has been shown to be rapid, increasing significantly up to 4 weeks, stable for at least 1 year (Gasmi et al., 2007b) and provides protection of nigral neurons against 6-OHDA-induced toxicity in a dose-dependent manner (Gasmi et al., 2007a). In aged monkeys receiving unilateral striatal injection of AAV2-NTN, there was robust expression of NTN and a significant increase in TH positive fibers in the striatum and an increase in the number of nigral TH positive cells 8 months post-administration (Herzog et al., 2007). In MPTP-treated hemiparkinsonian monkeys, administration of AAV2-NTN after lesioning produced long-lasting improvement in motor function within 1–3 months, persisting up to 10 months while also providing protection of nigral neurons and striatal DA fibers (Kordower et al., 2006).

Based on these positive results, an open label Phase I study was conducted to investigate the safety and tolerability of bilateral putamen injections of CERE-120 in 12 patients with advanced PD, resulting in no significant adverse effects, a significant decrease in UPDRS “off” scores, increase in “on” time without dyskinesias, and a reduction in “total off” time (Marks et al., 2008). Based on these findings a double-blind, sham surgery controlled Phase II trial was conducted in PD patients, though no changes in UPDRS “off” scores were found (Marks et al., 2010). However, post-mortem analysis of 2 subjects revealed deficits in retrograde transport of CERE-120 from the putamen to the SN (Bartus et al., 2011). This result led to a large Phase I/IIb trial investigating the effects of CERE-120 when administered both intraputaminally and intranigrally, with Phase I stage complete and CERE-120 being received without complications (Bartus et al., 2013). Data from the Phase IIb study was recently announced by Ceregene, with no significant differences between treatment groups in the primary endpoint as measured by UPDRS scores. However, according to the company, a significant difference was found in patient diary “off” scores, one of the key secondary endpoints (Ceregene, 2013). While the results of this trial are disappointing, they provide further evidence for the safety of CERE-120 and the use of gene therapy a viable strategy for the delivery of neurotrophic factors.

## The next stages of therapeutic development

It is clear that the therapeutics discussed in this review have emerged out of an increasingly well-grounded knowledge of the circuits underlying BG function and the role of neurotransmitters and neural regulation in these circuits (Figure 1). Consequently, while L-Dopa remains the mainstay of treatment, the major advances are coming from directing treatments to modify other aspects of BG circuit function. While many of these non-dopaminergic treatments may not erase the need for DA replacement strategies, they may support the use of these therapies through the reduction in L-Dopa dose, improvement of motor symptoms, and/or reducing LIDs.

We have only focused on the new wave of treatments that have shown promise in animal models and that have also gone to human clinical trials, however, efforts to identify ever more viable therapeutic targets continues. Notable developments in animal models in this area include histamine H3 antagonists which, in addition to their non-motor effects, are able to improve L-Dopa-induced motor effects (Nowak et al., 2008); dopamine uptake inhibitors, which potentiate the efficacy of L-Dopa (Huot et al., 2014); and conserved dopamine neurotrophic factor (CDNF), which has demonstrated neuroprotective properties and rescue of motor deficits (Ren et al., 2013). This, together with efforts directed to address the basic mechanisms and causes of PD, our increased understanding of genetics, and recent extensive efforts to develop new biomarkers that should in turn improve clinical trials, provides a great basis for optimism.

The next stage, in particular the development of neuroprotective drugs, will require advances in basic science, specifically, an understanding of the basic biology of PD including (1) the increasingly accepted role of non-neuronal cells in neurodegenerative diseases, (2) the underlying disease mechanisms (3) whether genetic mutations fit into multiple separate pathways that define different forms of the disease, or whether they fit in a single pathway that will ultimately elucidate the cause of PD and consequently, (4) consideration of PD as multifactorial and perhaps as several different diseases that manifest similarly, (5) advances in clinical trial design and interpretation, possibly assisted by biomarker measurements and (6) identification of early events and understanding the validity of Braak staging and its implications. We consider only some of these points briefly below.

### The role of genetics

Considerable advances have been made in the identification of genes associated with PD (Klein and Westenberger, 2012; Singleton et al., 2013), though further research remains to be conducted to further understand the relationship between genetic factors, PD onset, and clinical manifestation. These genetic studies have provided a basis for research into the molecular pathways underlying PD. However, it remains an issue that genetic penetrance varies, suggesting that mutant genes alone do not necessarily cause PD, indeed mutations account for approximately 30% of familial and 3–5% of sporadic PD cases (Klein and Westenberger, 2012). Furthermore, it is unclear if all mutations associated with PD risk cause a similar phenotype. Thus, it remains uncertain as to how applicable outcomes of molecular and biochemical studies of specific genes will be relevant to the PD populations more generally.

An important outcome of genetic studies is that they offer a potential approach to model aspects of human disease. Indeed, several transgenic mouse lines have now been developed that express mutant genes implicated in human PD. While these transgenic mice display anatomical and physiological abnormalities, many of them show only subtle phenotypes that are not associated with a loss of dopaminergic neurons, the traditional pathological hallmark of PD (Fleming et al., 2005; Melrose et al., 2006). Furthermore, the question of how broadly the specific models will apply to all of PD remains. For example, there is considerable interest in developing drugs that target leucine-rich repeat kinase-2 (LRRK2) activity, in its mutant form, a known genetic contributor to PD (Deng et al., 2012) and it seems likely that in such specific cases, the LRRK2 animal models could be useful for therapeutic testing. However, the general relevance of the LRRK2 animal models for PD is unclear. As such, it is unclear that these models will rapidly replace the chemical models that have shown such significant impact on understanding and treating PD to date.

### Consideration of PD etiology as multifactorial

Unlike the traditional view of a single disease state, it has been suggested that PD may be a collection of multiple diseases that share a common phenotype. It is unclear if each genetic, chemical and environmental form of the disease are all part of a common route to a single disease or alternatively, a series of different disease forms with a similar ultimate outcome. If the latter is true, then it seems unlikely that there will be an one-size-fits all therapy. It also raises questions as to whether clinical trials that include the entire spectrum of Parkinson's sufferers are necessarily the best sample population and if diagnosis and testing of treatments should ultimately include genetic testing and individualized therapies.

The low penetrance of PD and the variable involvement of alpha-synuclein and/or Lewy bodies suggests that many factors, in combination or as “multiple hits,” is required for the degeneration characteristic of PD (Sulzer, 2007). This multiple hit hypothesis argues that in addition to mechanisms such as oxidative stress, excitotoxicity, inflammation etc., multiple risk factors including genetic predisposition, toxin exposure, aging, and potentially other unknown factors, all interact to produce what we classify as PD.

This model explores the possibility that some forms of the disease may in fact have an early, possibly even prenatal, onset as a result of specific disruption to neuronal development. It has been suggested that early damage to the dopaminergic system via various developmental genes such as Nurr1 and Pitx3 could result in patients born with a lower number of DA neurons or a higher than normal vulnerability to a second environmental factor (Weidong et al., 2009). It is known that the nigrostriatal DA system is able to compensate for significant losses of DA neurons in the SN to allow the CNS to maintain normal motor function (Calne and Zigmond, 1991). In addition, DA neurons have evolved multiple mechanisms to protect themselves against cytosolic DA-related cellular stress from which it has been suggested their unique structure and function predisposes them to (Sulzer, 2007). Thus, when there are multiple insults, as suggested to occur in PD, the system can no longer regulate itself.

In line with the multi-hit hypothesis, genetic animal models of PD may represent early disease stages or an increase in vulnerability to secondary factors. Indeed, when coupled with environmental risk factors, including neurotoxins as used in PD research, these transgenic mouse lines often display marked neurodegeneration (Oliveira et al., 2003). Therefore the use of animal models that employ both genetics and toxins may be beneficial to the investigation of a disease that is fast becoming identified as one with a mutlifactorial etiology.

### Clinical trials

In order to identify more efficacious drugs for treating PD symptoms and/or halting neurodegeneration, advancements in clinical trial design will also be required, specifically, the way in which PD and its progression is diagnosed and monitored. The discovery of biomarkers that relate to specific disease stages will significantly aid in this area by allowing inclusion of more precisely defined patient populations in clinical trials and more accurate assessment of novel therapies against precise staging. Major efforts are underway in this area, but we note here that the same issue applies to biomarkers as to clinical trials generally with a one-size-fits-all disease marker likely hard to identify. Disease diversity may also need to be taken more strongly into consideration in clinical trials. Just as APOE4 genetic status is now used to stratify patients when analyzing clinical trials in Alzheimer's disease (Salloway et al., 2014), patients in PD trials may also need to be grouped genetically in post-hoc analyses. This may allow for the identification of subsets of patients in which the therapy may be showing the most promise.

Testing of neuroprotective agents directed to prevent disease progression will remain a particular challenge in light of these issues. It is likely that the efficacy of a given disease-modifying therapy in a given individual will depend on the specific disease cause and disease stage. It is a widely accepted problem that PD is not commonly diagnosed until approximately 80% of DA neurons are already lost and, if Braak staging (section Beyond the midbrain dopamine system) is generally correct, damage to other regions will have begun even before DA loss. It is therefore likely that achieving an unequivocal disease-modifying result against specific outcomes in a short 6–12 month trial in a population of PD patients, each with different disease causes and each at different disease stages, is going to remain difficult. The challenge is compounded by a lack of strategies for early disease detection, likely resulting in neuroprotective drugs being tested too late to induce significant benefit.

This became evident during the ADAGIO (Attenuation of Disease Progression with Azilect Given Once-Daily) clinical trial of rasagiline (Olanow et al., 2009), which utilized a complex “randomized start” design that has not truly been validated and furthermore assumes that the course of PD is fairly well defined. The outcome of that trial remains ambiguous, with a US Food and Drug Administration advisory committee voting unanimously against including a disease-modifying effect of rasagiline as one of its indications (Food and Drug Administration, 2011). Similarly, the Deprenyl and Tocopherol Antioxidative Therapy of Parkinsonism (DATATOP) study initially described the effects of selegiline as neuroprotective, only to be confounded later by the recognized symptomatic effects (Parkinson Study Group, 1989, 1993). These outcomes are likely to reflect, in part, all the issues outlined above, including challenges in identifying an unequivocal result in a relatively short trial and in such a diverse population.

In sum, until improvements in issues of disease diversity, stage, and monitoring are resolved it remains likely that current design of clinical trials to test both effective symptomatic and true disease-modifying therapies is going to remain a significant challenge.

### Beyond the midbrain dopamine system

Recent evidence has provided a framework in which the staging of PD can be reconsidered and therefore the validity of solely focusing on DA reassessed. Research conducted by Braak and colleagues (2002) demonstrated a correlation between insoluble alpha-synuclein deposition, its location, and the stage of the disease (Braak et al., 2002). They proposed that the neuropathological profile of PD progresses in a characteristic and non-random manner, and more importantly in a largely caudo-rostral direction over time (Braak et al., 2002, 2003, 2004). Known as Braak staging, six stages of neuropathology represent “pre-symptomatic” and “symptomatic” phases. In stages 1 and 2 alpha-synuclein deposits begin in the dorsal motor nucleus of the vagus nerve, olfactory bulb and medulla, with no noticeable motor symptoms. In stage 3 the pathology progresses from the brainstem but remains subcortical, with deposits found in the midbrain and basal forebrain. In stage 4, deposits are found in cortical regions and cell loss is evident in the SN. It is at this stage that individuals may display the first symptoms consistent with Parkinsonism. Finally, stages 5 and 6 show few remaining neuromelanin-positive cells in the SN and alpha-synuclein deposits begin to invade the neocortex, with patients displaying severe motor symptoms and cognitive dysfunction. It is important to note that the validity, accuracy and clinical relevance of Braak staging is controversial, with many critics publishing numerous studies describing individual differences in pathology and symptomology, indicating that not all cases fit into this caudo-rostral progression (Attems and Jellinger, 2008; Parkkinen et al., 2008; Jellinger, 2009). Regardless, the concept of a progression of degeneration and dysfunction that begins outside the dopaminergic system warrants significant thought and investigation. Furthermore, such staging not only allows for the development of biomarkers for the early detection of PD, in itself an extremely necessary avenue to pursue, but highlights the need to consider how animal models may recapitulate these stages. A recent study has provided a means for thinking about this issue (Luk et al., 2012).

Finally, there continues to be ever increasing understanding of the BG circuits that regulate movement. By using ever-refined methods and techniques the understanding of the circuit and the role of the major brain regions involved is being refined (Draganski et al., 2008; Kravitz et al., 2010; Redgrave et al., 2010; Ryczko et al., 2013). The consequence of this increased understanding of how the BG functions is going to inevitably lead to refined approaches to treatment.

### Hope for neuroprotective therapies

When examined as a whole, the treatment strategies outlined in this review display clinical outcomes that are skewed toward symptomatic relief of the motor symptoms of PD, or reducing LIDs. Clearly the next stage of therapeutic development is to identify disease-modifying treatments that offer neuroprotection. Progress in understanding the onset of PD has provided a number of candidate targets for neuroprotective and neurorescue interventions. As mentioned previously, the only potential disease-modifying agent identified in clinical trials to date is rasagiline, but the conflicting results and subsequent FDA analysis predicts that there will unlikely be clear evidence that rasagiline will be able to halt the progression of PD (Parkinson Study Group, 2004; Olanow et al., 2009). Nevertheless, results in animal models to date provide some promise that disease-modifying therapies may be emerging, in particular mGluR antagonists (section Metabotropic glutamate receptors), calcium channel antagonists (section Calcium channel blockers), and GLP-1 based therapeutics (section Glucagon-like peptide 1 (GLP-1) agonist). Finally, the role of inflammation (section Anti-inflammatories) offers an interesting avenue that will potentially reveal new therapeutics going forward. Meanwhile, it remains unclear, as to whether growth factor treatments, especially if delivered by gene therapy, will also offer promise (section Neurotrophic factors). It seems likely that much more needs to be understood of the specific mechanisms, processes and events underlying cell loss in PD before significant progress can be made in this area.

## Conclusions

The major advances in treating PD have come from our understanding of the mechanisms of the disease, much of which emerged from animal models. With the introduction of L-Dopa as a PD therapy approximately 50 years ago, the outlook and quality of life for patients dramatically changed and a number of potential treatments have since been identified. The new wave of treatments coming through in recent times has involved moving away from dopamine–mimetic treatments to a set of treatments that work in entirely new ways. Together with the extensive work in progress directed to both understand and treat the disease, we may be in the midst of a revolution in understanding and treating PD.

## Author contributions

Both Sandy Stayte and Bryce Vissel were responsible for all research, writing and editing of the manuscript.

## Sources of support

This work was supported by Bill Gruy; Iain S. Gray Foundation; Stanley and John Roth; David King; Doug Battersby; Geoff and Dawn Dixon; Geoffrey Towner; Tony and Vivian Howland-Rose; Walter and Edith Sheldon; Gleneagle Securities; Amadeus Energy Ltd.; Nick and Melanie Kell; Wicking Trust and the Mason Foundation; and SpinalCure Australia.

### Conflict of interest statement

The authors declare that the research was conducted in the absence of any commercial or financial relationships that could be construed as a potential conflict of interest.

## Abbreviations

AMPA: α-amino-3-hydroxy-5-methyl-4-isoxazolepropionic acid
MPP^+^: 1-methyl-4-phenylpyridinium
MPTP: 1-methyl-4-phenyl-1,2,3,6-tetrahydropyridine
DOPAC: 3,4-Dihydroxyphenylacetic acid
6-OHDA: 6-hydroxydopamine
AIMs: abnormal involuntary movements
AAV: adeno-associated virus
AADC: aromatic l-amino acid decarboxylase
ADAGIO: Attenuation of Disease Progression with Rasagiline Once-daily
BG: basal ganglia
Ca^2+^: calcium
CNS: central nervous system
CDNF: conserved dopamine neurotrophic factor
COX: cyclooxygenase
DATATOP: Deprenyl and Tocopherol Antioxidative Therapy of Parkinsonism
DA: dopamine
GDNF: glial derived neurotrophic factor
GPi: globus pallidus internus
GLP-1: glucagon-like peptide 1
GAD: glutamic acid-decarboxylase
HVA: homovanillic acid
LRRK2: leucine-rich repeat kinase-2
LIDs: L-Dopa-induced dyskinesias
LFADLDS: Lang-Fahn Activities of Daily Living Dyskinesia Scale
L-Dopa, LPS: levodopa; lipopolysaccharide
mGluRs: metabotropic glutamate receptors
NMDA: N-methyl-D-aspartate
NTN: neurturin
NSAIDs: non-steroidal anti-inflammatory drugs
NE: norepinephrine
PD: Parkinson's disease
ROS: reactive oxygen species
5-HT: serotonin
Na^+^: sodium
SN: substantia nigra
SNr: substantia nigra pars reticulata
STN: subthalamic nucleus
TH: tyrosine hydroxylase
UPDRS: Unified Parkinson's Disease Rating Scale
VMAT2: vesicular monoamine transporter 2.
