# Corrigendum: Advances in non-dopaminergic pharmacological treatments of Parkinson's disease

**DOI:** 10.3389/fnins.2014.00254

**Published:** 2014-08-19

**Authors:** Sandy Stayte, Bryce Vissel

**Affiliations:** ^1^Neuroscience, Neurodegenerative Disorders Laboratory, Garvan Institute of Medical ResearchSydney, NSW, Australia; ^2^Faculty of Medicine, University of New South WalesSydney, NSW, Australia

**Keywords:** L-dopa, Parkinson's disease, animal models, dopamine, dyskinesias, gene therapy, neurodegeneration, therapeutics

Figure 1 of the article by Stayte and Vissel (2014) contained an error during editing, which we now rectify. In the original Figure 1, the blue arrows representing the GABAergic projections to the LGP originate from the cerebral cortex. However, the blue arrows should be originating from the striatum. We include the updated version of Figure 1 with this correction.

**Figure 1:**
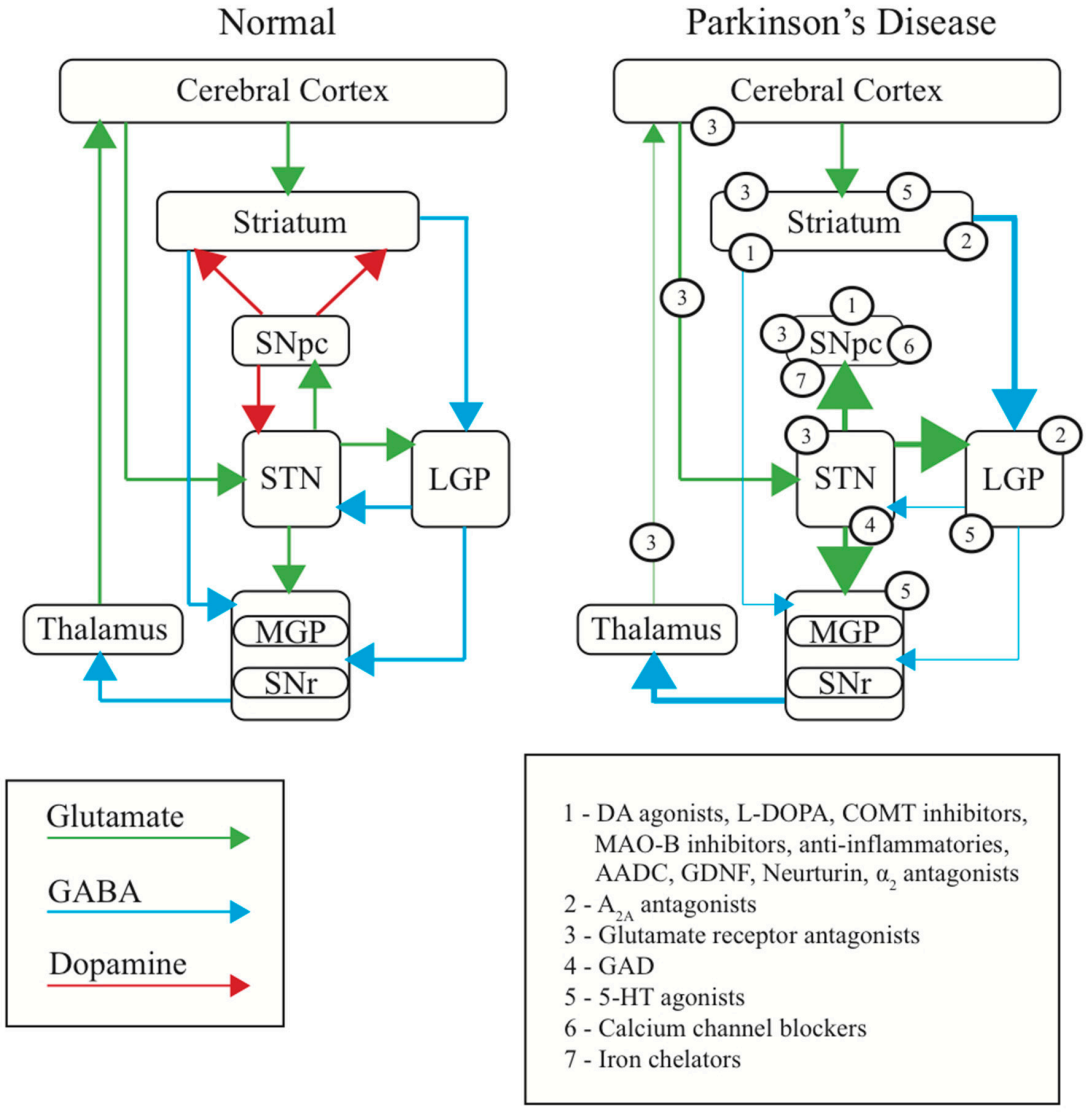
**Basal ganglia dysfunction in PD**. Diagram representing the normal function of the basal ganglia **(Left)**, the changes occurring in PD **(Right)**, and the site of primary action of therapeutic targets discussed in this review (numbered). Arrows represent the major neurotransmitters of glutamate (green), GABA (blue) and dopamine (red). Relative thickness of the arrows indicates level of activity of neurotransmitter. SNpc, substantia nigra pars compacta; SNr, substantia nigra reticulata; STN, subthalamic nucleus; MGP, medial globus pallidus; LGP, lateral globus pallidus.

## Conflict of interest statement

The authors declare that the research was conducted in the absence of any commercial or financial relationships that could be construed as a potential conflict of interest.
